# Effects of covid-19 pandemic on life expectancy and premature mortality in 2020: time series analysis in 37 countries

**DOI:** 10.1136/bmj-2021-066768

**Published:** 2021-11-03

**Authors:** Nazrul Islam, Dmitri A Jdanov, Vladimir M Shkolnikov, Kamlesh Khunti, Ichiro Kawachi, Martin White, Sarah Lewington, Ben Lacey

**Affiliations:** 1 Clinical Trial Service Unit and Epidemiological Studies Unit (CTSU), Nuffield Department of Population Health, Big Data Institute, University of Oxford, Oxford, UK; 2 Max Planck Institute for Demographic Research, Rostock, Germany; 3 International Laboratory for Population and Health, National Research University Higher School of Economics, Moscow, Russian Federation; 4 Diabetes Research Centre, University of Leicester, Leicester, UK; 5 NIHR Applied Research Collaboration–East Midlands, Leicester General Hospital, Leicester, UK; 6 Department of Social and Behavioral Sciences, Harvard T.H. Chan School of Public Health, Harvard University, Boston, MA, USA; 7 MRC Epidemiology Unit, University of Cambridge, Cambridge, UK; 8 MRC Population Health Research Unit, Nuffield Department of Population Health, University of Oxford, Oxford, UK

## Abstract

**Objective:**

To estimate the changes in life expectancy and years of life lost in 2020 associated with the covid-19 pandemic.

**Design:**

Time series analysis.

**Setting:**

37 upper-middle and high income countries or regions with reliable and complete mortality data.

**Participants:**

Annual all cause mortality data from the Human Mortality Database for 2005-20, harmonised and disaggregated by age and sex.

**Main outcome measures:**

Reduction in life expectancy was estimated as the difference between observed and expected life expectancy in 2020 using the Lee-Carter model. Excess years of life lost were estimated as the difference between the observed and expected years of life lost in 2020 using the World Health Organization standard life table.

**Results:**

Reduction in life expectancy in men and women was observed in all the countries studied except New Zealand, Taiwan, and Norway, where there was a gain in life expectancy in 2020. No evidence was found of a change in life expectancy in Denmark, Iceland, and South Korea. The highest reduction in life expectancy was observed in Russia (men: −2.33, 95% confidence interval −2.50 to −2.17; women: −2.14, −2.25 to −2.03), the United States (men: −2.27, −2.39 to −2.15; women: −1.61, −1.70 to −1.51), Bulgaria (men: −1.96, −2.11 to −1.81; women: −1.37, −1.74 to −1.01), Lithuania (men: −1.83, −2.07 to −1.59; women: −1.21, −1.36 to −1.05), Chile (men: −1.64, −1.97 to −1.32; women: −0.88, −1.28 to −0.50), and Spain (men: −1.35, −1.53 to −1.18; women: −1.13, −1.37 to −0.90). Years of life lost in 2020 were higher than expected in all countries except Taiwan, New Zealand, Norway, Iceland, Denmark, and South Korea. In the remaining 31 countries, more than 222 million years of life were lost in 2020, which is 28.1 million (95% confidence interval 26.8m to 29.5m) years of life lost more than expected (17.3 million (16.8m to 17.8m) in men and 10.8 million (10.4m to 11.3m) in women). The highest excess years of life lost per 100 000 population were observed in Bulgaria (men: 7260, 95% confidence interval 6820 to 7710; women: 3730, 2740 to 4730), Russia (men: 7020, 6550 to 7480; women: 4760, 4530 to 4990), Lithuania (men: 5430, 4750 to 6070; women: 2640, 2310 to 2980), the US (men: 4350, 4170 to 4530; women: 2430, 2320 to 2550), Poland (men: 3830, 3540 to 4120; women: 1830, 1630 to 2040), and Hungary (men: 2770, 2490 to 3040; women: 1920, 1590 to 2240). The excess years of life lost were relatively low in people younger than 65 years, except in Russia, Bulgaria, Lithuania, and the US where the excess years of life lost was >2000 per 100 000.

**Conclusion:**

More than 28 million excess years of life were lost in 2020 in 31 countries, with a higher rate in men than women. Excess years of life lost associated with the covid-19 pandemic in 2020 were more than five times higher than those associated with the seasonal influenza epidemic in 2015.

## Introduction

Since the emergence of SARS-CoV-2, health policy measures employed to minimise the impact of the covid-19 pandemic have varied substantially across countries and jurisdictions.^1^ ^2^ ^3^ ^4^ ^5^ ^6^ ^7^ These policy measures have affected many social and economic determinants of health,^8^ ^9^ ^10^ ^11^ including accessibility to healthcare services.^12^ ^13^ ^14^ ^15^ The overall impact of the pandemic and its associated policy measures therefore have implications for mortality beyond deaths with covid-19—the accuracy and completeness of which has been questioned in many countries and jurisdictions.^2^ ^16^

Data on all cause mortality are considered more reliable indicators of the impact of the covid-19 pandemic because they are less sensitive to coding errors, competing risks, and the potential for misclassification in designating the cause of deaths, and as such enable comparisons between countries.^17^ ^18^ ^19^ ^20^ ^21^ We have previously reported a large difference between reported deaths with covid-19 and estimated excess deaths associated with the covid-19 pandemic in 2020.^22^ Previous studies have used historical baseline mortality data over the recent past to estimate the expected number of deaths in 2020 and provide the basis for estimating excess deaths (observed minus expected deaths), which capture both the direct (deaths with covid-19) and the indirect (deaths from other causes) effects of the pandemic and associated policy measures.^18^ ^19^ ^20^ ^21^ ^22^ Although using excess deaths has been considered the ideal method for measuring the impact of the pandemic,^15^ this metric does not take into account age at death. When people die at an older age, they lose fewer years of remaining life.^23^ ^24^ Analysis of life expectancy and years of life lost (YLL) provide a more nuanced estimation of premature mortality at population level. Life expectancy, a widely used metric of mortality, is an indication of how long on average people can expect to survive if the age specific mortality rates of that year remain constant for the remainder of their life.^25^ ^26^ YLL takes into account the age distributions of mortality by giving greater weights to deaths that occur at younger ages.^24^ An important difference exists between life expectancy and YLL. Whereas life expectancy is a standardised measure based on a hypothetical life table cohort, YLL is calculated from the numbers of deaths observed in real populations. Therefore, life expectancy depends solely on mortality, and YLL (even after dividing by population size) depends on both the mortality and the age structure of the population.

Previous studies have reported the effects of the pandemic on reduction in life expectancy in the United States,^27^ ^28^ England and Wales,^29^ and Spain,^26^ largely based on partial data in 2020. Earlier studies have reported the YLL based on deaths with covid-19 only.^23^ ^30^ ^31^ ^32^ This method has several limitations because deaths with covid-19 were reported to have varying degrees of accuracy and incompleteness^2^ ^16^ ^23^; covid-19 mortality data are often not disaggregated by age and sex, which are required for the calculation of YLL^2^; and the impact of the pandemic and its associated policy measures on deaths from other causes are not captured.^15^ ^22^ A recent study by Aburto and colleagues examined changes in life expectancy between 2019 and 2020 in 29 developed countries and provided important information on differences between countries, including the best and the worst performers.^33^ However, this study was based on preliminary death statistics for reported countries and did not include data from Canada, Israel, Latvia, Luxembourg, New Zealand, Russia, South Korea, and Taiwan. The study did not report on the change in YLL either.

Most earlier studies compared life expectancy or YLL in 2020 with that in 2019 or an average of the most recent few years, which might lead to incorrect conclusions (see supplementary file).

In this study, we report the changes in life expectancy at birth and excess YLL from all causes in 2020 by comparing the observed life expectancy and YLL in 2020 with those that would be expected based on historical trends in 2005-19 in 37 high income countries.

## Methods

### Study design and eligibility

This study is a time series analysis of annual data on all cause mortality obtained from 37 upper-middle and high income countries with reliable, valid, and complete mortality data between 2005 and 2020 disaggregated by age and sex.

### Source of data

We obtained data from the Human Mortality Database, in which mortality and population data from authoritative national agencies are collated and standardised. The database is maintained by the Department of Demography at the University of California, Berkeley, US and the Max Planck Institute for Demographic Research, Rostock, Germany.^34^ ^35^ Mortality data for 2020 were obtained from the Short-term Mortality Fluctuations data series (a new extension of the Human Mortality Database).^36^ For the purposes of this study, we required annual mortality data to be disaggregated by age groups (<1, 1-4, 5-9, . . . 90-95, and ≥100) and sex.

Data for 2020 were available for 37 countries: Austria, Belgium, Bulgaria, Canada, Chile, Croatia, Czech Republic, Denmark, England and Wales, Estonia, Finland, France, Germany, Greece, Hungary, Iceland, Israel, Italy, Latvia, Lithuania, Luxembourg, the Netherlands, New Zealand, Northern Ireland, Norway, Poland, Portugal, Russia, Scotland, Slovakia, Slovenia, South Korea, Spain, Sweden, Switzerland, Taiwan, and the US. Details on the source and the methodology for collection and standardisation of data from each of these countries or regions have been published previously.^36^ ^37^ ^38^ In many countries, age groups originally available in Short-term Mortality Fluctuations data differed somewhat from the required granular age scale in this study. In six countries (Canada, Israel, Germany, New Zealand, South Korea, and the US) Short-term Mortality Fluctuations data included relatively coarse age groups (see supplementary file).

### Statistical analysis

#### Calculation of life expectancy and YLL in 2020 and 2015

Whenever possible, we used annual estimates from the Human Mortality Database based on official data (see supplementary file). Using the available data series in the Human Mortality Database starting from 2005, we employed the Lee-Carter model^39^ to extrapolate annual death rates, which consequently serve as the input to estimate age specific population exposures and expected death counts under the assumption of zero migration. Expected age specific death rates were predicted separately for men and women. We obtained expected death counts and population exposures for both men and women by summing the data for each. We used the forecasted population exposures and observed death counts from the Short-term Mortality Fluctuations data series to calculate observed death rates for countries where annual estimates for 2020 were not available from the Human Mortality Database.

Before calculating observed age specific death rates, we standardised the death counts from Short-term Mortality Fluctuations according to the International Organization for Standardization 8601-2004 guidelines^36^ and adjusted for incomplete weekly death statistics in 2020. The age and sex specific adjustment coefficients for incompleteness were calculated using the average annual ratio of the Short-term Mortality Fluctuations data (ie, sum of weekly death counts) to annual death counts during the past five years.

**Fig 1:**
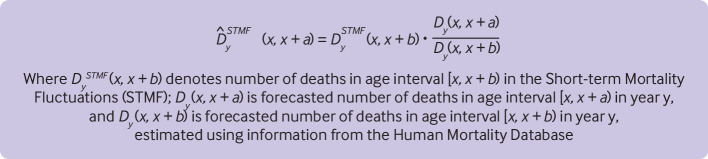
Equation for splitting aggregated age groups using distribution of forecasted death counts in the Human Mortality Database

In the absence of detailed mortality data by granular age groups, especially at young ages (eg, <1, 1-4 years) and old ages (eg, 85-89, 90-94 years) for 2020 in Short-term Mortality Fluctuations, we split aggregated age groups using distribution of forecasted death counts from the Human Mortality Database (fig 1). Details of the methodology have been published previously.^36^ We checked the accuracy of life expectancy estimates depending on granular or broad age intervals in the Short-term Mortality Fluctuations data and found only small deviations, even for broad age scales (see supplementary file for details of the methodology, including sensitivity analysis, and supplementary figures S1 and S2). For 2015 data, we used annual death counts and population exposures by five year age groups from the Human Mortality Database.

We derived life expectancy from abridged life tables, which were constructed using standard life table methodology.^40^ ^41^ The supplementary file provides details of the methodology.

**Fig 2:**
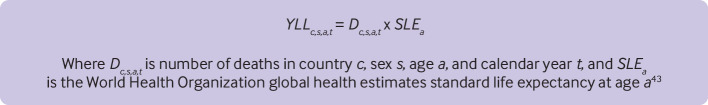
Equation used to estimate years of life lost (YLL)

To attribute an equal lifetime loss produced by a death at the same age across the countries,^42^ ^43^ we calculated the YLL from the World Health Organization standard life table using the methodology developed by the Global Burden of Disease, Injuries and Risk Factor study.^43^ ^44^ The equation in figure 2 was used to estimate the YLL.

#### Calculation of changes in life expectancy at birth and YLL in 2015 and 2020

Within each country, sex, and age groups, the reduction in life expectancy was calculated as the difference between the observed and expected life expectancy in 2020. The expected life expectancy for 2020 was based on Lee-Carter forecasting using observed 2005-19 data.^39^ Similarly, the expected YLL was computed for 2020, and excess YLL was calculated as the difference between observed and expected YLL within each country, sex, and age group. The sum of the excess YLL across the age groups, separately by sex, was used to estimate country specific total excess YLL and excess YLL (per 100 000 population) in 2020. As recommended, the excess YLL estimates were rounded to three significant digits to avoid spurious accuracy.

We estimated statistical uncertainty using a bootstrap method. Following a standard demographic approach, we did not calculate confidence intervals for life expectancy and YLL at the national level. Thus, we considered the mortality forecast as the only source of statistical uncertainty. The confidence intervals were based on the sample of 5000 iterations generated: firstly, we derived a distribution of age specific forecasted mortality rates and then we generated a random set of age specific death rates and calculated our variables of interest (life expectancy, YLL, and changes in life expectancy and YLL in 2020). The 2.5th quantile and the 97.5th quantile of the bootstrap distribution for each statistic were used as the 95% confidence intervals. The procedure was applied independently to each country and sex strata.

Our reference period for predicting mortality in 2020 is longer than the period of 2015-19 used in several earlier studies. The period 2015-19 includes substantial increases in mortality during the winters of 2015, 2017, and 2018 that contributed to an attenuation of the mortality improvements in many developed countries in these years.^45^ ^46^ ^47^ Consequently, the choice of 2015-19 as a reference period might result in artificially increased baseline mortality levels and underestimation of losses in life expectancy and excess YLLs in 2020.

To put our findings into context, we also calculated the change in life expectancy and the YLL associated with the seasonal influenza epidemic in 2015 following the same methodology using data between 2000 and 2014 as the reference period. We chose 2015 as a comparator to 2020 because in 2015 the 37 countries under study experienced the smallest average annual improvement in mortality among all years between 2005 and 2019, coinciding with a noticeable increase in mortality during winter.^45^ ^48^ ^49^

#### Decomposition of life expectancy losses in the US, Lithuania, Poland, and Spain

Earlier research^25^ ^33^ including our preliminary analysis showed the highest life expectancy losses in the US compared with other OECD (Organisation for Economic Co-operation and Development) countries in 2020. Our previous study on excess mortality, however, reported the highest excess crude death rates in Lithuania, Poland, Spain, Hungary, Italy, Belgium, Slovenia, England and Wales, and Czech Republic, followed by the US.^22^ To explain these important discrepancies, we conducted an exploratory decomposition analysis of the life expectancy losses of 2020 in the US, and three countries with highest excess crude death rates in 2020 (Lithuania, Poland, and Spain) using the Andreev-Arriaga-Pressat method.^50^ ^51^ ^52^ ^53^

Statistical analyses were done using R (version 4.1.0) in RStudio. The Lee-Carter forecast was performed using the R package *demography*.^54^

### Patient and public involvement

Patients and the public were not involved in this study because of the ongoing covid-19 pandemic.

## Results

### Changes in life expectancy in 2020

**Fig 3:**
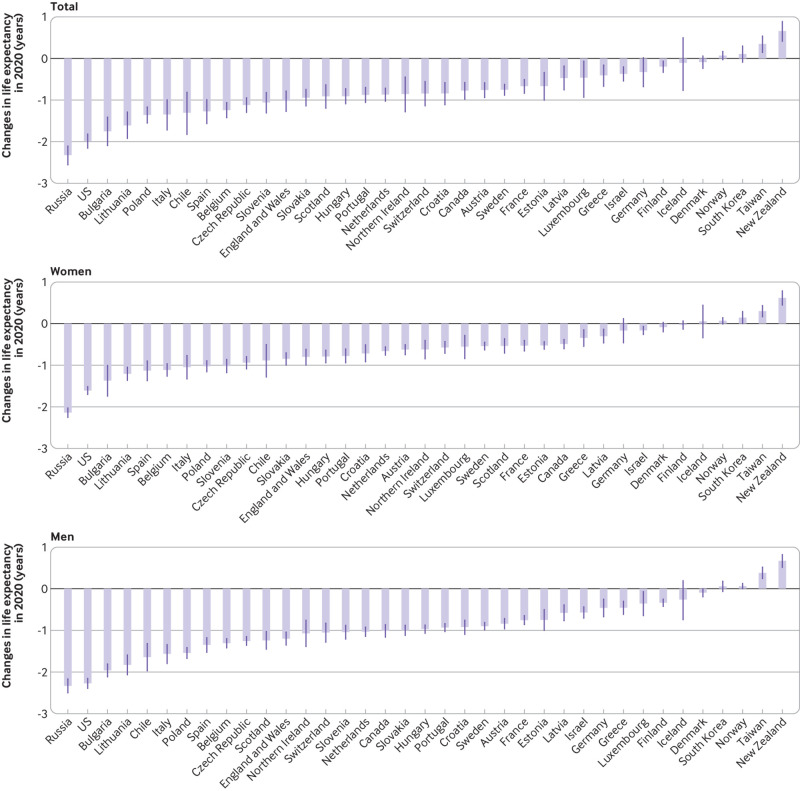
Changes in life expectancy at birth associated with covid-19 pandemic in 2020. Change is calculated as the difference between observed and expected life expectancy, estimated using the Lee-Carter model^39^

In all the countries between 2005 and 2019, an increasing trend was observed in life expectancy at birth, both in men and women (supplementary figure S3). However, most countries showed a reduction in life expectancy in 2020, with the largest overall reduction in life expectancy at birth (in years) in Russia (−2.32, 95% confidence interval −2.55 to −2.11), the US (1.98, −2.16 to 1.82), Bulgaria (−1.75, −2.09 to −1.41), Lithuania (−1.61, −1.92 to −1.29), and Poland (−1.36, −1.55 to −1.17). Reductions in life expectancy in Italy, Spain, and England and Wales were −1.35 (−1.72 to −0.99), −1.27 (−1.57 to −0.99), and −1.02 (−1.27 to −0.78), respectively. In contrast, a gain in life expectancy was observed in New Zealand (0.66, 0.41 to 0.89) and Taiwan (0.35, 0.14 to 0.54); no evidence was found of a change in life expectancy in South Korea (0.11, −0.09 to 0.30), Norway (0.07, −0.03 to 0.17), or Denmark (−0.09, −0.24 to 0.06) (fig 3).

In all countries but Luxembourg, men had a higher reduction in life expectancy at birth than women. The reduction in life expectancy in men was highest in Russia (−2.33, −2.50 to −2.17), the US (−2.27, −2.39 to −2.15), Bulgaria (−1.96, −2.11 to −1.81), Lithuania (−1.83, −2.07 to −1.59), and Chile (−1.64, −1.97 to −1.32). In women, the reduction in life expectancy was highest in Russia (−2.14, −2.25 to −2.03), the US (−1.61, −1.70 to −1.51), Bulgaria (−1.37, −1.74 to −1.01), Lithuania (−1.21, −1.36 to −1.05), and Spain (−1.13, −1.37 to −0.90) (fig 3 and supplementary table S2).

### Changes in years of life lost in 2020

**Fig 4:**
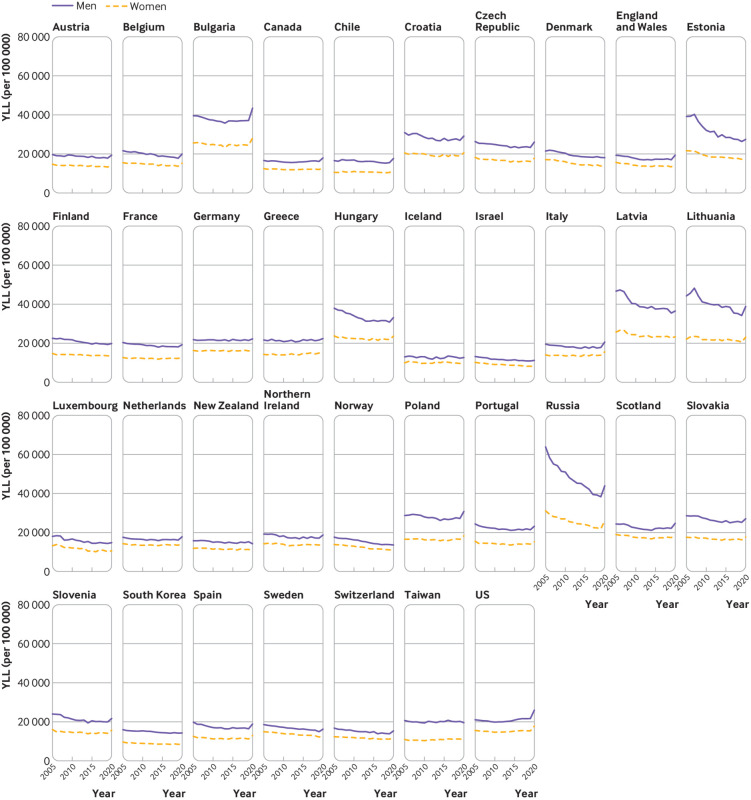
Years of life lost (YLL) per 100 000 during 2005-20

Years of life lost declined in most countries in both men and women between 2005 and 2019, except Canada, Greece, Scotland, Taiwan, and the US (fig 4). The observed YLL in 2020 was higher than expected in all countries except Taiwan and New Zealand, where there was a reduction in YLL, and Iceland, South Korea, Denmark, and Norway, where there was no evidence of a change in YLL in 2020. In the remaining 31 countries, more than 222 million (130 million in men and 92.6 million in women) years of life were lost in 2020, which is 28.1 million (95% confidence interval 26.8m to 29.5m) YLL higher than expected. The excess YLL in men and women were 17.3 million (16.8m to 17.8m) and 10.8 million (10.4m to 11.3m), respectively.

**Fig 5:**
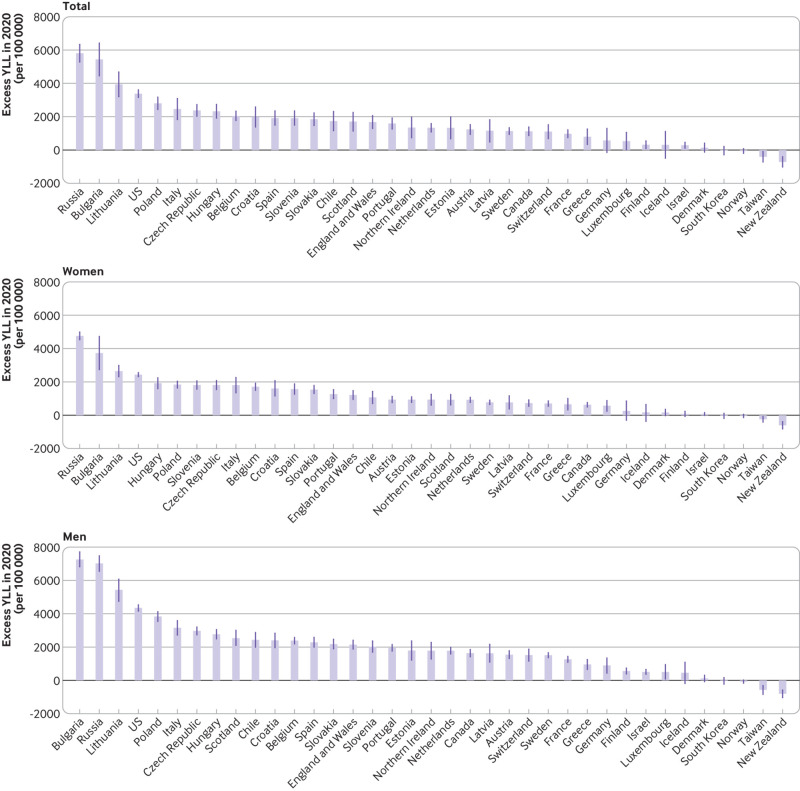
Excess years of life lost (YLL) in 2020 (per 100 000). Change is calculated as the difference between observed and expected life expectancy estimated using Lee-Carter model^39^

In men and women combined, excess YLL (per 100 000) were highest in Russia (5810, 95% confidence interval 5280 to 6340), Bulgaria (5440, 4460 to 6420), Lithuania (3940, 3200 to 4680), the US (3380, 3160 to 3610), and Poland (2800, 2430 to 3170), with a higher rate in men than women. The highest excess YLL per 100 000 in men were observed in Bulgaria (7260, 6820 to 7710), Russia (7020, 6550 to 7480), Lithuania (5430, 4750 to 6070), the US (4350, 4170 to 4530), and Poland (3830, 3540 to 4120); excess YLL in women were highest in Russia (4760, 4530 to 4990), Bulgaria (3730, 2740 to 4730), Lithuania (2640, 2310 to 2980), the US (2430, 2320 to 2550), and Hungary (1920, 1590 to 2240) (fig 5 and supplementary table S3).

**Fig 6:**
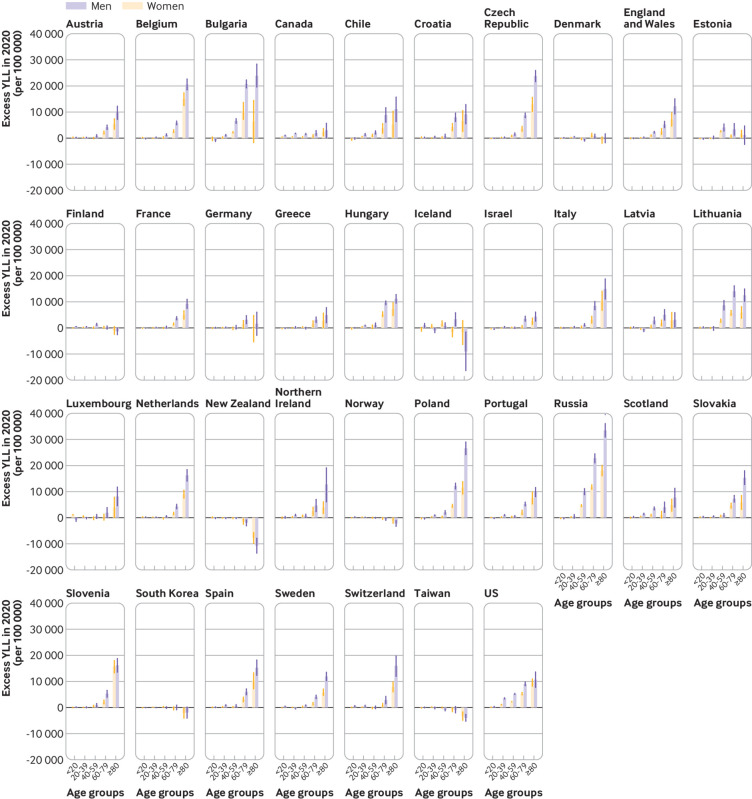
Excess years of life lost (YLL) (per 100 000) by age and sex in 2020

Supplementary figure S4 shows the trend of YLL during 2005-20 by age and sex. Figure 6 shows the excess YLL in 2020 by age and sex. In general, excess YLL increased with age, both in men and women. However, Finland, Iceland, New Zealand, South Korea, and Taiwan had lower than expected YLL in the elderly population (≥80 years). These countries had a small increase, or a decrease, in YLL in other age groups as well (fig 6). Excess YLL rate was generally lower in people younger than 65 years, except in Russia (3290, 2780 to 3810), Bulgaria (2650, 2220 to 3070), Lithuania (2580, 1790 to 3410), and the US (2390, 2280 to 2510), with excess YLL rate >2000 per 100 000. The ratio of YLL rate between people aged <65 and ≥65 years was 0.2 or higher in Estonia, Canada, Scotland, the US, Lithuania, and Chile (see supplementary table S4).

### Age components of life expectancy losses in US and comparator countries, 2020

**Fig 7:**
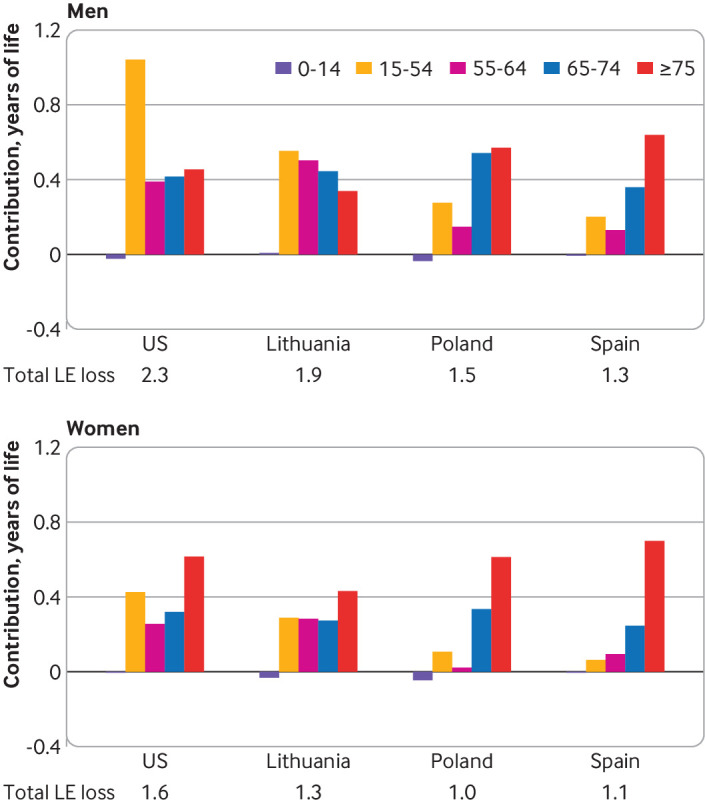
Age group components of difference between observed and expected life expectancy (LE) in 2020 among men and women in the United States and three comparator countries with the highest excess crude death rates in 2020. *Highest excess death rates according to Islam et al.^22^ The life table decomposition analysis was conducted using the Andreev-Arriaga-Pressat method^50^ ^51^ ^52^ ^53^

Figure 7 shows age components of the life expectancy losses in the US, Lithuania, Poland, and Spain in men and women produced by differences between the observed and expected age specific death rates by age intervals 0-14, 15-54, 55-64, 65-74, and ≥75. In the US and Lithuania it appears that mortality excess in people younger than 65 years, particularly among men, was responsible for a high proportion of the total losses in life expectancy: the respective values for men and women were 62% and 42% in the US and 58% and 44% in Lithuania. Corresponding values in Poland were 27% and 8% and in Spain were 26% and 15%. These two countries, especially Spain, showed an expected pattern, with the dominating role of older ages as a driver of losses in life expectancy.

### Changes in life expectancy and years of life lost: covid-19 (2020) *v* influenza epidemic (2015)

**Fig 8:**
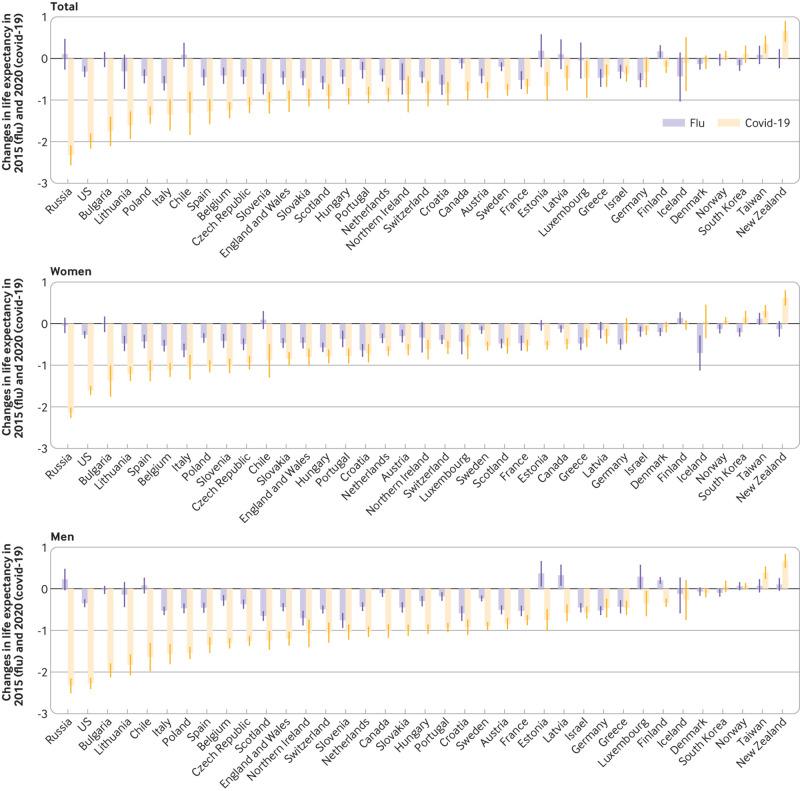
Changes in life expectancy during covid-19 pandemic in 2020 compared with seasonal influenza epidemic in 2015

**Fig 9:**
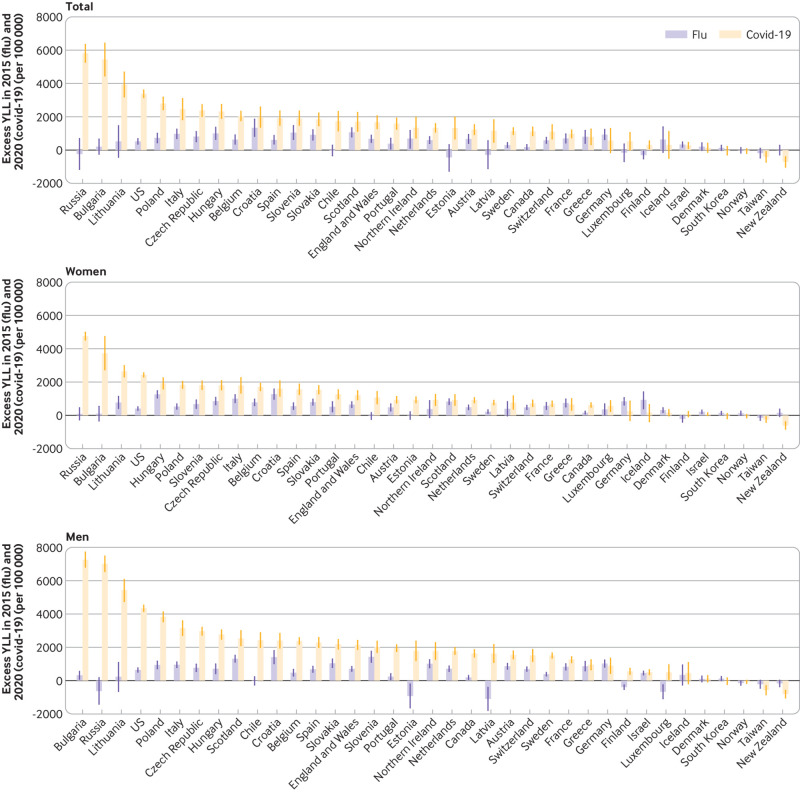
Changes in years of life lost (YLL) (per 100 000) during covid-19 pandemic in 2020 compared with seasonal influenza epidemic in 2015

Most countries experienced a reduction in life expectancy in 2015, but the reduction in 2020 was substantially greater than that in 2015. Most countries had excess YLL during the seasonal influenza epidemic in 2015, except Chile, Estonia, Luxembourg, Latvia, Finland, New Zealand, Russia, and Taiwan (fig 8 and fig 9). The rate of excess YLL was, however, much higher in most countries during the covid-19 pandemic in 2020 compared with the seasonal influenza epidemic in 2015. Overall, the excess YLL in the 37 countries was 5.5 times higher during the covid-19 pandemic (2510 per 100 000, 95% confidence interval 2390 to 2630 per 100 000) in 2020 than the excess YLL associated with the seasonal influenza epidemic in 2015 (458, 325 to 592), with an absolute difference of 2050 years of life lost per 100 000.

## Discussion

In this global comparative study of 37 countries, a reduction in life expectancy was found in men and women in all countries except New Zealand, Taiwan, and Norway, where there was a gain in life expectancy in 2020. We found no evidence of a change in life expectancy in 2020 in Denmark, Iceland, and South Korea. The highest reduction in life expectancy in men was observed in Russia, the US, Bulgaria, Lithuania, and Chile; the highest reduction in women was observed in Russia, the US, Bulgaria, Lithuania, and Spain. Years of life lost were higher than expected in all countries except Taiwan, New Zealand, Iceland, Denmark, South Korea, and Norway. In the remaining 31 countries, about 28 million excess years of life were lost in 2020 (17 million in men and 11 million in women). The highest excess YLL in both men and women were observed in Russia, Bulgaria, Lithuania, the US, and Poland. The excess YLL rate was relatively low in people younger than 65 years, except in Russia, the US, Lithuania, and Bulgaria. The excess YLL rates associated with the covid-19 pandemic in 2020 were more than five times higher than those associated with the seasonal influenza epidemic in 2015.

### Comparison with previous literature

Country specific estimates of life expectancy, largely based on partial data in 2020, have been reported for the US,^27^ ^28^ England and Wales,^29^ and Spain.^26^ In the US, the trajectory of life expectancy at birth during 2010-20 reported in our study is similar to that reported by the Centers for Disease Control and Prevention (CDC).^27^ Drawing on data from the first half of 2020, this study reported a life expectancy of 77.8 years in the US,^27^ whereas the estimated life expectancy at birth in our study was 77.4 years. Similarly, our estimates of life expectancy at birth were 74.6 years in men and 80.3 years in women, which was slightly lower than those reported in the CDC study (75.1 and 80.5 years, respectively).^27^ These differences could be due to a varying timeline used to estimate life expectancy—the CDC study used data up to June 2020, whereas we used data for the full year. Moreover, our earlier study showed that the excess death rates in the younger age groups (15-64 years) increased during the latter months in 2020 (especially during October-December).^22^

In Aburto and colleagues’ study of England and Wales, using data up to the 47th week (ending 20 November) of 2020, the estimated life expectancy was 82.6 years in women and 78.7 years in men.^29^ These findings are almost identical to the estimates from our study (82.7 and 78.7 years, respectively).

Using data up to 5 July 2020, a previous study reported an estimated life expectancy of 79.5 and 85.0 years in men and women, respectively, in Spain in 2020.^26^ These estimates are almost identical to those from our study (79.6 and 85.0 years in men and women, respectively).

Multi-country life expectancy estimates and analyses of life expectancy losses across countries have recently been published.^55^ In particular, the Eurostat has published preliminary estimates of life expectancy at birth for member countries of the European Union. Comparison between these estimates and our estimates was possible for 26 countries. For 21 countries, the differences were 0.1 years or lower (in both men and women), which is attributable to different methods for building abridged life tables and some remaining incompleteness of mortality data. The largest deviations (0.3 years) were seen in the small populations of Iceland, Latvia, and Luxembourg as well as Finland. However, the updated life expectancy estimates by Statistics Finland are similar to our estimates.^56^

A previous analysis reported the change in life expectancy across 29 developed countries.^33^ Our analysis includes eight additional countries. We were able to include data from Canada, Israel, South Korea, New Zealand, Luxembourg, and Latvia because we applied a more efficient procedure for estimation of the detailed mortality age distributions that fully exploited all available data from the Human Mortality Database. Taiwan was included because updates to Short-term Mortality Fluctuations allowed us to fill former data gaps. Russia was included because Russian data for 2020 has recently been included in Short-term Mortality Fluctuations. Data for these additional eight countries allowed us to observe most of the range of losses in life expectancy in developed countries as well as to highlight the favourable situation in New Zealand, Taiwan, and South Korea. For the subset of 29 countries present in both the studies, the life expectancy losses are in a good agreement with a Spearman’s correlation coefficient of 0.94 between the two rankings.

Most of the studies that reported on YLL used deaths with covid-19 to estimate potential YLL.^23^ ^30^ ^31^ ^32^ One earlier study examined the change in YLL due to excess deaths from all causes in 19 developed countries.^23^ All these studies, however, used country specific remaining life expectancies, and in this sense the estimated YLL are not comparable across the countries. Since deaths with covid-19 might result in an underestimation or, in some cases, overestimation of the overall impact of the pandemic on deaths and YLL, our estimates are not directly comparable to these estimates. One study estimated 20.5 million YLL in 81 countries based on projected deaths with covid-19,^23^ whereas our study estimated 28.1 million YLL in only 31 countries. These findings suggest a substantial underestimation of the overall impact on premature mortality if the estimates are based solely on deaths with covid-19 or when the estimation of YLL uses country specific life tables, or both. Moreover, covid-19 mortality data are often not disaggregated by age at the levels (eg, five year age categories) required for an accurate estimation of life expectancy and premature deaths.

With a much lower reported number of deaths with covid-19 (n=1613) in Lithuania, the estimated excess YLL were higher than in most of the countries (except Bulgaria and Hungary), as were the estimated excess deaths in a previous study.^22^ Our study found that the additional years of life lost associated with the covid-19 pandemic in 2020 were higher than those associated with the seasonal influenza epidemic in 2015, which is consistent with a previous report.^32^ Our finding of comparable or lower than expected YLL in 2020 in Taiwan, New Zealand, Iceland, and South Korea could be attributed to the successful pandemic elimination policies of these countries, including evidence based population health interventions.^57^ ^58^ ^59^ ^60^ ^61^ ^62^ Taiwan and New Zealand also had lower than expected YLL during the seasonal influenza epidemic in 2015. Exploring the precise reasons for this is beyond the scope of this study but could potentially be related to policy interventions, including seasonal influenza vaccine coverage and systemic resilience of the public health policy instruments.^3^ ^63^ Public health policy interventions aimed at reducing the transmission of SARS-CoV-2^1^ might have had other indirect effects (eg, a reduction in deaths from other causes, such as influenza and other respiratory infections, air pollution, road traffic incidents) contributing to an overall reduction in YLL in 2020.

### Strengths and limitations of this study

In addition to using the two major public health measures reflecting prematurity of death and accounting for trends, a key strength of our study was use of validated and standardised mortality data from authoritative national agencies to ensure comparability across countries and time. Rather than relying on deaths with covid-19, we used all cause mortality data in our analysis, which are less sensitive to coding error and misclassification in attributing the cause of deaths. We also estimated the expected YLL based on 15 years of historical data and applied a validated analytical approach that enables a more effective use of available data from the Human Mortality Database, which in turn allowed us to include more countries in the analysis. Rather than using an arbitrary age threshold, we used WHO standard life expectancy to enable international comparison of YLL following the standard methodology in the Global Burden of Disease, Injuries and Risk Factor study.^43^ ^44^ Our study does, however, have some limitations. Firstly, our study was restricted to countries with reliable data for the whole study period of 2000-20. Therefore, we did not include most countries from Asia, Africa, and Latin America. Our assumption of a no migration for population projection, when applicable, might not be generalisable to analyses at subnational levels. We also could not examine the variation in excess YLL by other critically important factors, such as socioeconomic status and race or ethnicity.^64^ ^65^ ^66^ ^67^ ^68^ ^69^ ^70^ ^71^ Previous studies also reported regional variability in reduction in life expectancy, such as in Spain,^26^ but we did not have detailed regional data to examine this important heterogeneity. Our study only reports the extent of premature lives lost in 2020. As of October 2021, however, the covid-19 pandemic is not yet over, and therefore future studies should estimate the long term burden of the pandemic.

### Policy implications and future directions

Our findings extend the existing literature on the direct and indirect effects of the covid-19 pandemic and associated policy measures.^22^ Our results strongly justify a more nuanced estimation of the lives lost beyond excess mortality. For example, with a similar burden of excess deaths per 100 000 in Spain and the US (161 and 160, respectively),^22^ excess YLL (per 100 000) was substantially higher in the US (3400) than in Spain (1900), indicating higher numbers of deaths at younger ages in the US compared with Spain.^22^ Indeed, the ratio of YLL rate in people aged <65 and ≥65 years at death was 0.29 in the US, whereas it was only 0.07 in Spain. Despite a lower excess death rate than Lithuania, Poland, and Spain,^22^ the reduction in life expectancy in the US was higher than in these three countries. A full examination of this phenomenon is beyond the scope of the current study. Nevertheless, the decomposition analysis of the life expectancy losses in these four countries reveals particularly large contributions to the reduction of life expectancy from increases in mortality at ages younger than 65 years in the US. However, our analyses were not able to identify whether these excess deaths were directly caused by SARS-CoV-2 or were related to other causes of deaths. The highest reduction in life expectancy, and highest increase in YLL, largely occurred in countries where the baseline life expectancy was relatively low. Therefore, baseline health status could have contributed to these results. Widespread ethnic inequality in the US, as reported previously, might have contributed to high YLL in the US.^25^ Future studies should conduct an in-depth examination to disentangle these factors.

Our findings of a comparable or lower than expected YLL in Taiwan, New Zealand, Denmark, Iceland, Norway, and South Korea underscore the importance of successful viral suppression and elimination policies, including targeted and population based public health policy interventions.^57^ ^58^ ^59^ ^60^ ^61^ ^62^ A comprehensive pandemic preparedness aimed at more resilient health systems could be key to tackling the impact of future pandemics.^3^ ^63^ Quantifying the effects of specific policy interventions on the reduction of premature deaths will help inform future policy intervention. As many of the effects of the pandemic might take a longer time frame to have a measurable effect on human lives, continuous and timely monitoring of excess YLL would help identify the sources of excess mortality and excess YLL in population subgroups.^72^

**What is already known on this topic**

Reported numbers of deaths with covid-19 are subject to changes within and across countries as well as some degrees of delays, inaccuracy, and incompleteness
Excess deaths (difference between observed and expected numbers of deaths from all causes) allows the assessment of the full impact of the pandemic, including the direct effect on deaths with covid-19, and the indirect effect of the pandemic on deaths from other diseases
Estimation of excess deaths does not, however, consider the age at death, and therefore does not quantify the impact of the pandemic on premature deaths as years of life lost (YLL)

**What this study adds**

In 2020, life expectancy was lower and YLL higher than expected in all countries except New Zealand, Taiwan, Iceland, South Korea, Denmark, and Norway—in the remaining 31 countries, >28 million excess years of life were lost
Highest reduction in life expectancy in 2020 was observed in Russia (men, −2.33 years; women, −2.14), the US (men, −2.27; women, −1.61), Bulgaria (men −1.96; women, −1.37), Lithuania (men, −1.83; women, −1.21), Chile (men, −1.64; women, −0.88), and Spain (men, −1.35; women, −1.13)
Excess YLL rates associated with the covid-19 pandemic in 2020 were more than five times higher than those associated with the seasonal influenza epidemic in 2015

## Data Availability

All the data used in this study are publicly available and properly cited. However, we plan to add the analytical codes available on a publicly available repository for reproducibility. More guided instruction to get access to the data for transparency and reproducibility will be provided on request made to the corresponding author at nazrul.islam@ndph.ox.ac.uk

## Web extra

Extra material supplied by authors

## Notes

Contributors: NI conceived the study with input from the coauthors. MW, SL, and BL are the co-senior authors. NI and DAJ conducted the statistical analysis. NI wrote the first draft. All the authors provided critical scholarly feedback on the manuscript. All the co-authors approved of the final version of the manuscript. NI and DAJ are the guarantors. The corresponding author attests that all listed authors meet authorship criteria and that no others meeting the criteria have been omitted.” at the end of the statement.

The study guarantors (NI and DAJ) affirm that the manuscript is an honest, accurate, and transparent account of the study being reported; that no important aspects of the study have been omitted; and that any discrepancies from the study as planned (and, if relevant, registered) have been explained.

Dissemination to participants and related patient and public communities: We will disseminate the findings to members of the public through press releases, institutional websites, and repositories, as well as personal communications, and social communication platforms. We also plan to write a BMJ Opinion article to describe it in more general terms for the members of the public.

Provenance and peer review: Not commissioned; externally peer reviewed.
