# Supplementary material for: Effects of covid-19 pandemic on life expectancy and premature mortality in 2020: time series analysis in 37 countries
Source: BMJ. 2021 Nov 3;375:e066768. doi: 10.1136/bmj-2021-066768 (PMC8564739; doi:10.1136/bmj-2021-066768)
Supplement: Supplementary file 1 — Supplementary information: additional information, tables, and figures [file isln066768.ww.pdf]

Effects of Covid-19 pandemic on life expectancy and premature mortality in 2020: time series analysis in 37 countries

Supplementary Materials

Table of Contents

Supplemental data availability ... 2

Supplementary methods: calculation of life expectancy ... 4

Panel-I: Graphical presentation of potential incorrect conclusion on excess mortality in the context of an increasing or decreasing trend ... 6

Supplementary Table S1. Data availability in the Human Mortality Database as of August 2021 ... 7

Supplementary Figure S1. The difference in life expectancy in 2020 by age between estimates based on original granular STMF data and estimates based on (first aggregated and then) re-distributed death counts for five countries with detailed original data. ... 8

Supplementary Figure S2. The difference in life expectancy in 2020 at ages 0, 15, 45, and 80 calculated using original granular data and life expectancy based on (first coarsely aggregated and then) re-distributed death counts. ... 9

Supplementary Table S2: Observed, expected and changes in life expectancy in 2020 in 37 high income countries ... 10

Supplementary Table S3: Observed, expected and changes in the years of life lost (per 100,000) in 2020 in 37 high income countries ... 13

Supplementary Table S4: Years of life lost per 100,000 in people <65 compared with people ≥65 years ... 16

Supplementary Figure S3: Life expectancy at birth, 2005-2020 ... 18

Supplementary Figure S4: Years of life lost (per 100,000) by age and sex, 2005-2020 ... 19

Fig. S4(A): Men ... 19

Fig. S4(B): Women ... 20

Supplemental data availability

We used data from the Human Mortality Database (HMD, www.mortality.org). For 2015, all calculations are based on annual data series that are available at the finest detail level: by one-year age group and open age interval 110+. Nevertheless, we used a 5-year age groups scale with an open age interval of 100+ (i.e., <1, 1-4, 5-9, ..., 95-99, 100+) for the sake of comparability to 2020 estimates.

For 2020, we used annual series in all cases where respective data were available (see Table S1). Belgium, Denmark, Finland, Norway, Portugal, and Russia had such extended data series. Data for the remaining 31 countries were completed using weekly death counts from the Short-Term Mortality Fluctuations data series (STMF, www.mortality.org) and population exposures based on Lee-Carter forecast as described in the Methods section. The standardized STMF data is available only by a broad age group 0-14, 15-64, 65-74, 75-84, 85+. Nevertheless, the STMF also provides the original death counts classified (in many cases) using more detailed age groups. Note, the age scale in the original data varies across countries (see Table S1, last column).

Most of the countries are using 5-year age groups but with the first age group from 0–4, instead of 0, and 1–4. Infant mortality is crucially important by the life table calculation. There are also six countries – Canada, Germany, Israel, New Zealand, South Korea, and the USA – with broad age groups (Table S1). There is no method for ungrouping coarsely aggregated data designed for the specific case of weekly death counts with elevated mortality due to pandemics.

The pattern of the COVID-19 deaths is very consistent across different settings in age groups below 65. [1] but excess mortality is often disproportionally concentrated at the old ages.[2,3] Thus, aggregated age groups were split using proportions from annual forecasted age-specific death counts (see details in the Methods section). To check the sensitivity of results to using the aggregated data, we do the following check.

First, we tested the importance of the first (infant mortality) and last age groups. For this analysis, we calculated life tables using five countries (England and Wales, France, Italy, Russia, and Scotland) with detailed data including infant mortality. Then, we aggregated the data in the first two age groups (<1 and 1–4) into one age group of 0–4, and made an open age interval of 90+. Using our standard method, we re-distributed aggregated age groups and re-calculated the life tables.

The difference in the estimated life expectancy produced by these two methods is presented in Figure S1, which shows that the difference between these two approaches was <0.01 for all ages below 80. Therefore, our approach of ungrouping coarsely aggregated data is robust.

Second, we tested the robustness of our methods for coarse age groups reported in Canada, Germany, Israel, New Zealand, South Korea, and the USA (Table S1). For this analysis, we used original data from the other 31 countries that had data by 5-year groups, and calculated the life tables. Then, we aggregated the data according to the age groups of the six countries with coarse age groups, and applied our method of ungrouping to re-calculate the life tables.

The boxplot (Figure S2) shows that the maximum difference produced by data aggregation is below 0.2 with the median close to zero; third and second quartiles are within the range of ±0.1. As expected, the biggest differences are observed in case of using most aggregated age scale (New Zealand, age groups 0-64, 65-79, 80+). At the same time, using age group 0-29 (age scale Germany) has only minor influence on life expectancy. Thus, the 2020 life expectancies calculated from STMF mortality data by aggregated age groups are virtually the same compared to LEs calculated from the originally granular STMF data.

Supplementary methods: calculation of life expectancy

First, we converted the death rates, ${}_nm_x$, into probabilities of death, ${}_nq_x$. Here, index n refers to the length of the interval, x denotes the beginning of the age interval. For example, ${}_nq_x$ denotes probability of death in age interval $[x, x+n)$.

Let, ${}_na_x$ be the average number of years lived within the age interval $[x, x+n)$ for people dying at that age. We assume that $a_x = \frac{n}{2}$ for all single-year ages except age 0 (see below). We then compute ${}_nq_x$ from ${}_nm_x$ and ${}_na_x$ according to the formula,

$$ {}_nq_x = \frac{{}_nm_x}{1+(1-{}_na_x)\cdot {}_nm_x} \quad (1) $$

for $x = 0, 1, 5, 10, \dots, 95$. For the open age interval (100+), we set ${}_\infty a_{100} = \frac{1}{{}_\infty m_{100}}$ and ${}_\infty q_{100} = 1$.

For infants <1 year of age, we used the formulas for a_0 suggested by Preston *et al.*,[4] which are adapted from the Coale-Demeny model life tables.[5] Thus, if $m_0 \geq 0.107$:

$$ a_0 = \begin{cases} 0.350 & \textit{for women} \\ 0.330 & \textit{for men} \end{cases} \quad (2) $$

On the other hand, if $m_0 < 0.107$:

$$ a_0 = \begin{cases} 0.053 + 2.800 \cdot m_0 & \textit{for women} \\ 0.045 + 2.684 \cdot m_0 & \textit{for men} \end{cases} \quad (3) $$

To complete the life table calculation, let ${}_np_x$ be the probability of surviving from age x to $x+n$. Therefore,

$$ {}_np_x = 1 - {}_nq_x \quad (4) $$

for all ages x. Let the radix (the starting number of new-borns) of the life table be $l_0 = 100{,}000$. Then, the number of survivors (out of 100,000) at age x is

$$ l_x = l_0 \cdot \prod_{i=0}^{i<x} {}_np_i \quad (5) $$

The distribution of deaths by age in the life-table population is

$$ {}_nd_x = l_x \cdot {}_nq_x \quad (6) $$

for $x = 0, 1, 5, \dots, 95$. For the open age category, ${}_\infty \mathrm{d}_{x100} = l_{100}$.

The person-years lived by the life-table population in the age interval $[x, x+n)$ are

$$ {}_nL_x = l_x - (1 - {}_na_x) \cdot {}_nd_x \quad (7) $$

for $x = 0, 1, 5, \dots, 95$. For the open age category, ${}_\infty \mathrm{L}_{100} = l_{100} \cdot a_{100}$. The person-years remaining for individuals of age x equal

$$T_x = \sum_{i=x}^{95} L_i + {}_{\infty}L_{100} \tag{8}$$

for $x = 0, 1, 5, \dots, 95.$ Remaining life expectancy at age *x* is

$$e_x = \frac{T_x}{l_x} \tag{9}$$

for $x = 0, 1, 5, \dots, 95.$

Confidence intervals for the forecasted life expectancy and YLL were calculated using the approach proposed by Chiang.[6] This approach requires known distribution of age-specific probabilities of death. We derived these distributions using confidence intervals for mortality rates returned by Lee-Carter forecast. For the Lee-Carter forecast and calculation respective confidence limits for mortality rates we used R package demography.

Panel-I: Graphical presentation of potential incorrect conclusion on excess mortality in the context of an increasing or decreasing trend

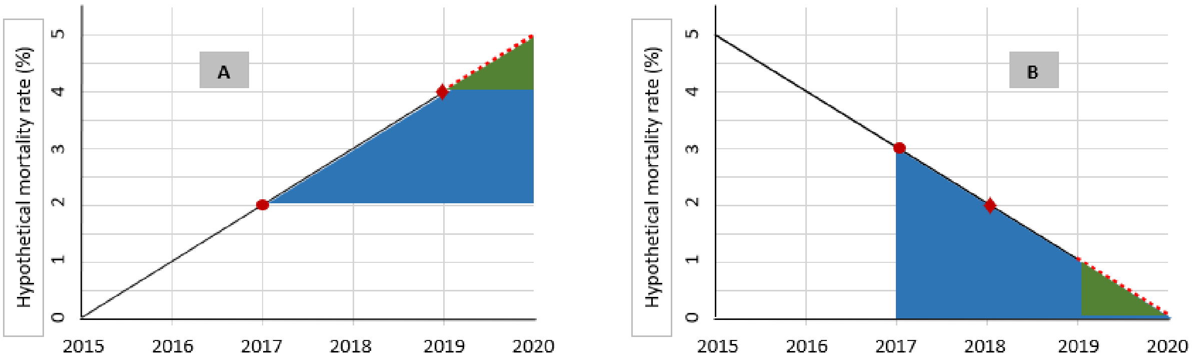

Panel I: (A) shows an increasing trend of a hypothetical mortality rate (%) from 0 to 4 between 2015 and 2019. Therefore, the most reasonable ‘expected’ value in 2020 will be approximately 5 (red dotted line). However, if the observed value in 2020 is >2 but <5 (in the blue or green shaded area), and if we compare this with the 2015-2019 average value of 2, we will incorrectly conclude that the observed value in 2020 was higher than the expected. If the observed value in 2020 is >4 but <5 (green-shaded area), we may incorrectly conclude that the observed was higher than expected if we compared with the 2019 value of 4. An analogous example of a decreasing trend is shown in (B). The reasonable expected value in 2020 is 0 (re dotted line), while the 2015-2019 average is 3. Therefore, any observed value of >0 & <3 would lead to an incorrect conclusion that there was a decrease in 2020. Similarly, any observed value >0 & <1 would lead to an incorrect conclusion that there was a decrease in 2020 if we compared this with the 2019 estimate of 1. Both of these examples illustrate how we can make an incorrect conclusion if we ignore the recent temporal trend (e.g., a gradual improvement in life expectancy over the last decades).

Supplementary Table S1. Data availability in the Human Mortality Database as of August 2021

Country	Last year in the HMD core	STMF age scale in 2020
Austria	2019	0-4, 5-9, ..., 85-89, 90+
Belgium	2020	0-4, 5-9, ..., 85-89, 90+
Bulgaria	2017	0-4, 5-9, ..., 85-89, 90+
Canada	2018	0-44, 45-64, 65-84, 85+
Chile	2017	0-4, 5-9, ..., 85-89, 90-94, 95+
Croatia	2019	0-4, 5-9, ..., 85-89, 90+
Czech Republic	2019	0-4, 5-9, ..., 85-89, 90+
Denmark	2020	0-4, 5-9, ..., 85-89, 90-94, 95-99, 100+
England and Wales	2018	0, 1-4, 5-9, ..., 85-89, 90+
Estonia	2019	0-4, 5-9, ..., 85-89, 90+
Finland	2020	0-4, 5-9, ..., 85-89, 90+
France	2019	0, 1-4, 5-9, ..., 85-89, 90-94, 95+
Germany	2017	0-29, 30-34, 35-39, ..., 90-94, 95+
Greece	2019	0-4, 5-9, ..., 85-89, 90+
Hungary	2019	0-4, 5-9, ..., 85-89, 90+
Iceland	2018	0-4, 5-9, ..., 85-89, 90+
Israel	2019	0-19, 20-29, ..., , 70-79, 80+
Italy	2018	0, 1-4, 5-9,..., 95-99, 100
Latvia	2019	0-4, 5-9, ..., 85-89, 90+
Lithuania	2019	0-4, 5-9, ..., 85-89, 90+
Luxembourg	2019	0-4, 5-9, ..., 85-89, 90+
Northern Ireland	2018	0-4, 5-9, ..., 85-89, 90+
Netherlands	2019	0-4, 5-9, ..., 85-89, 90+
New Zealand	2019	0-64, 65-79, 80+
Norway	2020	0-4, 5-9, ..., 85-89, 90-94, 95-99, 100+
Poland	2019	0-4, 5-9, ..., 85-89, 90+
Portugal	2020	0-4, 5-9, ..., 85-89, 90+
Russia	2020	0, 1-4, 5-9,..., 90-94, 95+
South Korea	2018	0,15,65,75,85
Scotland	2018	0, 1-4, 5-9,..., 90-94, 95+
Slovakia	2019	0-4, 5-9, ..., 85-89, 90+
Slovenia	2019	0-4, 5-9, ..., 85-89, 90+
Spain	2018	0-4, 5-9, ..., 85-89, 90+
Sweden	2019	0-4, 5-9, ..., 85-89, 90+
Switzerland	2018	0-4, 5-9, ..., 85-89, 90+
Taiwan	2019	0-4, 5-9, ..., 85-89, 90-94, 95-99, 100+
USA	2019	0, 1-4, 5-14, 15-24,...,75-84,85+

HMD core: Human mortality core database (www.mortality.org). STMF: Short-Term Mortality Fluctuations data series (STMF, www.mortality.org)

Supplementary Figure S1. The difference in life expectancy in 2020 by age between estimates based on original granular STMF data and estimates based on (first aggregated and then) re-distributed death counts for five countries with detailed original data.

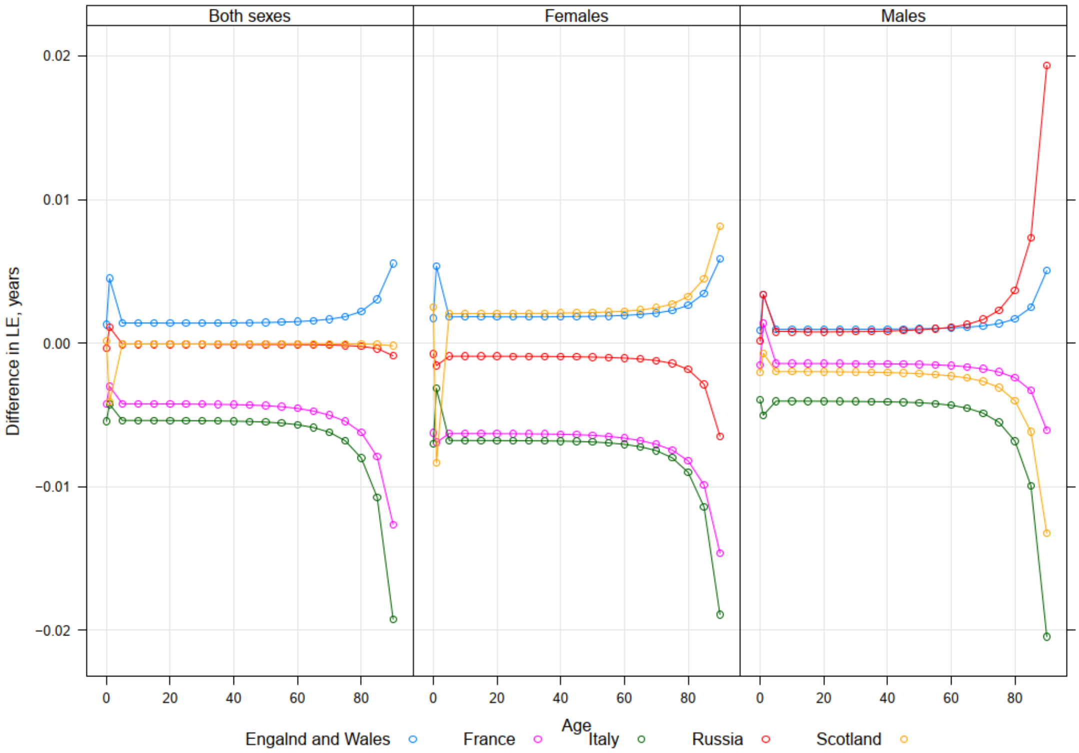

Notes: Original granular age groups: 0, 1-4, 5-9, 10-14, …, 100+
Simulated age scale: 0-4, …, 84-85, 90+

Supplementary Figure S2. The difference in life expectancy in 2020 at ages 0, 15, 45, and 80 calculated using original granular data and life expectancy based on (first coarsely aggregated and then) re-distributed death counts.

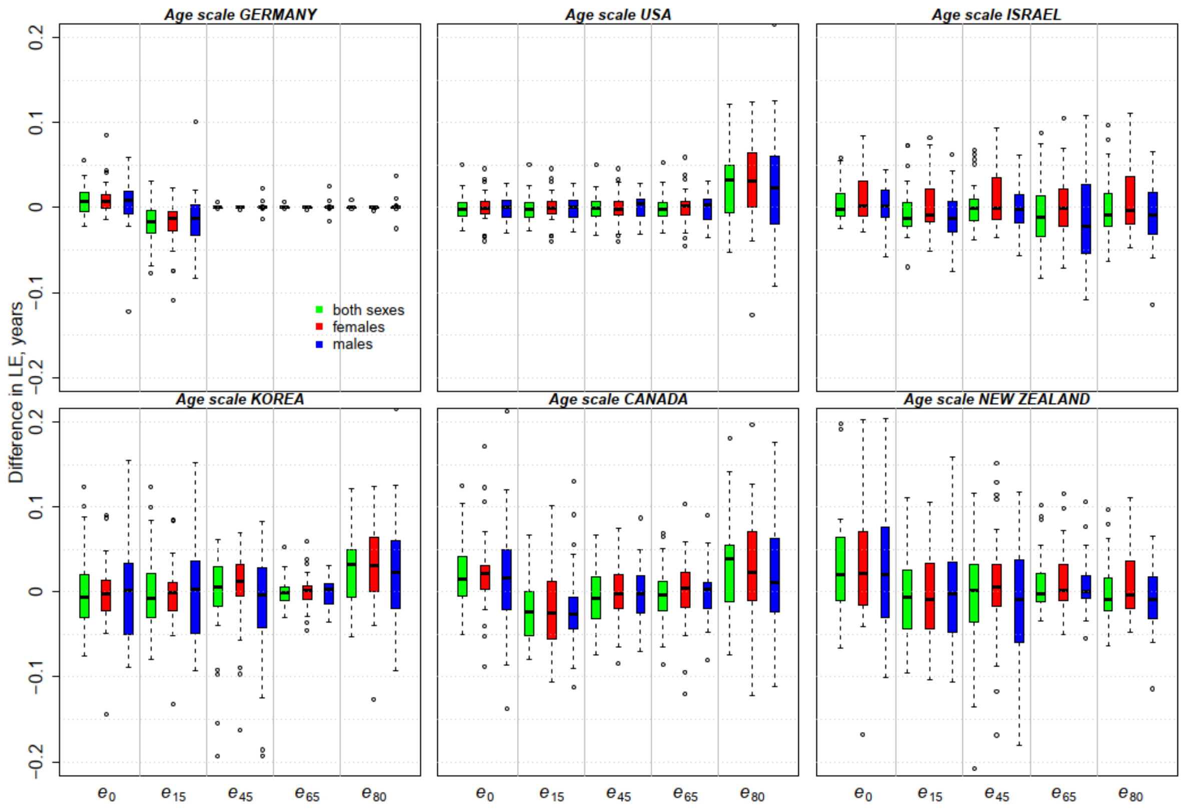

Notes: Simulated age scales are named after countries that use them (see Table S1).
Calculations on STMF data for 31 countries with the originally granular data in STMF: Austria, Belgium, Bulgaria, Chile, Croatia, Czechia, Denmark, England and Wales, Estonia, Finland, France, Greece, Hungary, Iceland, Italy, Latvia, Lithuania, Luxembourg, Northern Ireland, Netherlands, Norway, Poland, Portugal, Russia, Scotland, Slovakia, Slovenia, Spain, Sweden, Switzerland, Taiwan.

Supplementary Table S2: Observed, expected and changes in life expectancy in 2020 in 37 high income countries

Country	Sex	Observed	Expected (95% CI)	Difference (95% CI)
Austria	Total	81.32	82.08 (81.9 to 82.26)	-0.76 (-0.94 to -0.59)
Austria	Female	83.7	84.33 (84.21 to 84.45)	-0.62 (-0.75 to -0.51)
Austria	Male	78.91	79.75 (79.62 to 79.87)	-0.84 (-0.96 to -0.72)
Belgium	Total	80.78	82.02 (81.84 to 82.2)	-1.24 (-1.42 to -1.06)
Belgium	Female	83.03	84.14 (83.99 to 84.29)	-1.11 (-1.26 to -0.96)
Belgium	Male	78.52	79.82 (79.72 to 79.93)	-1.31 (-1.42 to -1.2)
Bulgaria	Total	73.53	75.28 (74.94 to 75.62)	-1.75 (-2.09 to -1.41)
Bulgaria	Female	77.42	78.79 (78.42 to 79.16)	-1.37 (-1.74 to -1.01)
Bulgaria	Male	69.91	71.87 (71.72 to 72.03)	-1.96 (-2.11 to -1.81)
Canada	Total	81.61	82.38 (82.18 to 82.58)	-0.77 (-0.98 to -0.58)
Canada	Female	83.89	84.38 (84.27 to 84.49)	-0.49 (-0.6 to -0.38)
Canada	Male	79.34	80.35 (80.2 to 80.5)	-1.01 (-1.16 to -0.86)
Switzerland	Total	83.06	83.91 (83.62 to 84.2)	-0.84 (-1.14 to -0.55)
Switzerland	Female	85.04	85.61 (85.47 to 85.75)	-0.57 (-0.71 to -0.43)
Switzerland	Male	81.04	82.09 (81.87 to 82.32)	-1.05 (-1.28 to -0.83)
Chile	Total	80.08	81.39 (80.9 to 81.9)	-1.31 (-1.82 to -0.81)
Chile	Female	83.05	83.94 (83.56 to 84.34)	-0.88 (-1.28 to -0.5)
Chile	Male	77.03	78.67 (78.34 to 78.99)	-1.64 (-1.97 to -1.32)
Czech Republic	Total	78.33	79.45 (79.27 to 79.62)	-1.12 (-1.3 to -0.95)
Czech Republic	Female	81.36	82.29 (82.15 to 82.44)	-0.94 (-1.09 to -0.79)
Czech Republic	Male	75.33	76.58 (76.48 to 76.69)	-1.26 (-1.36 to -1.15)
Germany	Total	81.02	81.35 (81.01 to 81.7)	-0.33 (-0.68 to 0.02)
Germany	Female	83.43	83.6 (83.31 to 83.89)	-0.17 (-0.46 to 0.12)
Germany	Male	78.62	79.08 (78.87 to 79.29)	-0.46 (-0.67 to -0.25)
Denmark	Total	81.55	81.64 (81.49 to 81.79)	-0.09 (-0.24 to 0.06)
Denmark	Female	83.51	83.6 (83.48 to 83.71)	-0.09 (-0.2 to 0.03)
Denmark	Male	79.58	79.68 (79.58 to 79.77)	-0.09 (-0.19 to 0)
England & Wales	Total	80.63	81.65 (81.41 to 81.9)	-1.02 (-1.27 to -0.78)
England & Wales	Female	82.62	83.42 (83.24 to 83.62)	-0.8 (-0.99 to -0.62)
England & Wales	Male	78.65	79.85 (79.69 to 80)	-1.2 (-1.35 to -1.04)
Spain	Total	82.29	83.56 (83.28 to 83.85)	-1.27 (-1.57 to -0.99)
Spain	Female	85.01	86.13 (85.9 to 86.38)	-1.13 (-1.37 to -0.9)
Spain	Male	79.55	80.9 (80.73 to 81.08)	-1.35 (-1.53 to -1.18)
Estonia	Total	78.47	79.13 (78.81 to 79.47)	-0.66 (-1 to -0.34)
Estonia	Female	82.45	82.97 (82.88 to 83.06)	-0.52 (-0.61 to -0.43)
Estonia	Male	74.1	74.85 (74.6 to 75.1)	-0.75 (-1 to -0.5)
Finland	Total	81.85	82.05 (81.92 to 82.19)	-0.2 (-0.33 to -0.07)
Finland	Female	84.62	84.66 (84.56 to 84.76)	-0.03 (-0.13 to 0.06)
Finland	Male	79.07	79.41 (79.32 to 79.49)	-0.34 (-0.42 to -0.25)
France	Total	82.23	82.9 (82.74 to 83.07)	-0.67 (-0.84 to -0.51)
France	Female	85.18	85.71 (85.58 to 85.84)	-0.53 (-0.66 to -0.4)

France	Male	79.2	79.95 (79.84 to 80.06)	-0.75 (-0.86 to -0.64)
Greece	Total	81.2	81.61 (81.36 to 81.87)	-0.41 (-0.67 to -0.16)
Greece	Female	83.74	84.08 (83.89 to 84.29)	-0.34 (-0.55 to -0.15)
Greece	Male	78.63	79.09 (78.93 to 79.25)	-0.46 (-0.61 to -0.3)
Croatia	Total	77.77	78.61 (78.35 to 78.88)	-0.84 (-1.11 to -0.58)
Croatia	Female	80.88	81.59 (81.39 to 81.79)	-0.72 (-0.92 to -0.51)
Croatia	Male	74.66	75.58 (75.42 to 75.76)	-0.92 (-1.09 to -0.75)
Hungary	Total	75.73	76.64 (76.46 to 76.82)	-0.91 (-1.09 to -0.73)
Hungary	Female	79.03	79.82 (79.67 to 79.97)	-0.79 (-0.94 to -0.64)
Hungary	Male	72.32	73.29 (73.19 to 73.39)	-0.97 (-1.07 to -0.87)
Iceland	Total	82.85	82.95 (82.35 to 83.61)	-0.11 (-0.77 to 0.5)
Iceland	Female	84.41	84.36 (83.97 to 84.75)	0.05 (-0.34 to 0.44)
Iceland	Male	81.33	81.59 (81.13 to 82.07)	-0.26 (-0.74 to 0.19)
Israel	Total	82.76	83.13 (82.96 to 83.3)	-0.37 (-0.54 to -0.2)
Israel	Female	84.77	84.93 (84.84 to 85.03)	-0.16 (-0.26 to -0.07)
Israel	Male	80.66	81.23 (81.09 to 81.36)	-0.57 (-0.7 to -0.43)
Italy	Total	82.15	83.49 (83.14 to 83.87)	-1.35 (-1.72 to -0.99)
Italy	Female	84.45	85.5 (85.22 to 85.78)	-1.05 (-1.33 to -0.77)
Italy	Male	79.78	81.34 (81.12 to 81.57)	-1.56 (-1.8 to -1.34)
South Korea	Total	83.73	83.62 (83.43 to 83.82)	0.11 (-0.09 to 0.3)
South Korea	Female	86.59	86.45 (86.3 to 86.59)	0.14 (0 to 0.29)
South Korea	Male	80.63	80.57 (80.45 to 80.7)	0.06 (-0.06 to 0.18)
Lithuania	Total	75.08	76.69 (76.37 to 77)	-1.61 (-1.92 to -1.29)
Lithuania	Female	80.02	81.22 (81.06 to 81.38)	-1.21 (-1.36 to -1.05)
Lithuania	Male	70.05	71.87 (71.64 to 72.11)	-1.83 (-2.07 to -1.59)
Luxembourg	Total	82.07	82.53 (82.13 to 83)	-0.46 (-0.93 to -0.07)
Luxembourg	Female	84.35	84.9 (84.64 to 85.19)	-0.55 (-0.84 to -0.28)
Luxembourg	Male	79.81	80.17 (79.88 to 80.46)	-0.35 (-0.64 to -0.07)
Latvia	Total	75.43	75.9 (75.61 to 76.18)	-0.47 (-0.76 to -0.18)
Latvia	Female	79.94	80.24 (80.07 to 80.4)	-0.3 (-0.46 to -0.14)
Latvia	Male	70.61	71.19 (71 to 71.38)	-0.58 (-0.77 to -0.39)
Northern Ireland	Total	80.14	81 (80.59 to 81.43)	-0.86 (-1.28 to -0.45)
Northern Ireland	Female	82.02	82.64 (82.42 to 82.87)	-0.62 (-0.85 to -0.4)
Northern Ireland	Male	78.23	79.3 (78.99 to 79.62)	-1.07 (-1.39 to -0.76)
Netherlands	Total	81.36	82.23 (82.07 to 82.38)	-0.87 (-1.03 to -0.72)
Netherlands	Female	83.03	83.69 (83.59 to 83.79)	-0.66 (-0.76 to -0.56)
Netherlands	Male	79.67	80.7 (80.59 to 80.81)	-1.03 (-1.14 to -0.92)
Norway	Total	83.2	83.13 (83.03 to 83.23)	0.07 (-0.03 to 0.17)
Norway	Female	84.87	84.81 (84.73 to 84.88)	0.07 (-0.01 to 0.14)
Norway	Male	81.5	81.43 (81.37 to 81.49)	0.06 (0.00 to 0.13)
New Zealand	Total	82.68	82.02 (81.79 to 82.26)	0.66 (0.41 to 0.89)
New Zealand	Female	84.44	83.82 (83.65 to 83.99)	0.62 (0.45 to 0.79)
New Zealand	Male	80.86	80.19 (80.04 to 80.35)	0.67 (0.51 to 0.82)
Poland	Total	76.76	78.12 (77.93 to 78.31)	-1.36 (-1.55 to -1.17)
Poland	Female	80.88	81.9 (81.77 to 82.03)	-1.02 (-1.16 to -0.89)
Poland	Male	72.76	74.3 (74.17 to 74.43)	-1.54 (-1.67 to -1.41)

Portugal	Total	81.04	81.92 (81.74 to 82.1)	-0.88 (-1.06 to -0.7)
Portugal	Female	83.98	84.75 (84.58 to 84.92)	-0.78 (-0.94 to -0.61)
Portugal	Male	77.93	78.86 (78.77 to 78.96)	-0.93 (-1.03 to -0.84)
Russia	Total	71.48	73.8 (73.59 to 74.04)	-2.32 (-2.55 to -2.11)
Russia	Female	76.36	78.5 (78.39 to 78.61)	-2.14 (-2.25 to -2.03)
Russia	Male	66.45	68.78 (68.62 to 68.95)	-2.33 (-2.5 to -2.17)
Scotland	Total	78.45	79.36 (79.09 to 79.65)	-0.91 (-1.2 to -0.63)
Scotland	Female	80.72	81.26 (81.09 to 81.43)	-0.54 (-0.71 to -0.36)
Scotland	Male	76.18	77.42 (77.2 to 77.63)	-1.24 (-1.45 to -1.03)
Slovakia	Total	76.97	77.92 (77.72 to 78.11)	-0.94 (-1.14 to -0.75)
Slovakia	Female	80.36	81.21 (81.07 to 81.36)	-0.85 (-1.00 to -0.70)
Slovakia	Male	73.53	74.53 (74.4 to 74.65)	-1.00 (-1.12 to -0.88)
Slovenia	Total	80.55	81.61 (81.37 to 81.86)	-1.06 (-1.31 to -0.82)
Slovenia	Female	83.39	84.41 (84.25 to 84.57)	-1.02 (-1.18 to -0.86)
Slovenia	Male	77.76	78.8 (78.64 to 78.97)	-1.04 (-1.21 to -0.88)
Sweden	Total	82.46	83.21 (83.08 to 83.34)	-0.75 (-0.88 to -0.62)
Sweden	Female	84.31	84.85 (84.75 to 84.94)	-0.54 (-0.63 to -0.45)
Sweden	Male	80.66	81.56 (81.47 to 81.64)	-0.90 (-0.99 to -0.81)
Taiwan	Total	81.18	80.83 (80.64 to 81.04)	0.35 (0.14 to 0.54)
Taiwan	Female	84.34	84.05 (83.91 to 84.18)	0.3 (0.17 to 0.43)
Taiwan	Male	78.13	77.75 (77.61 to 77.88)	0.38 (0.25 to 0.52)
United States	Total	77.28	79.27 (79.1 to 79.44)	-1.98 (-2.16 to -1.82)
United States	Female	80.21	81.82 (81.73 to 81.91)	-1.61 (-1.7 to -1.51)
United States	Male	74.44	76.71 (76.59 to 76.83)	-2.27 (-2.39 to -2.15)

Supplementary Table S3: Observed, expected and changes in the years of life lost (per 100,000) in 2020 in 37 high income countries

Country	Sex	Observed	Expected (95% CI)	Difference (95% CI)
Austria	Total	16700	15500 (15200 to 15800)	1230 (941 to 1520)
Austria	Female	14200	13200 (13000 to 13400)	936 (755 to 1130)
Austria	Male	19400	17800 (17600 to 18100)	1540 (1310 to 1780)
Belgium	Total	17600	15500 (15200 to 15800)	2040 (1770 to 2330)
Belgium	Female	15300	13500 (13300 to 13800)	1710 (1490 to 1920)
Belgium	Male	20000	17600 (17400 to 17800)	2390 (2190 to 2580)
Bulgaria	Total	35500	30100 (29100 to 31000)	5440 (4460 to 6420)
Bulgaria	Female	28100	24400 (23400 to 25400)	3730 (2740 to 4730)
Bulgaria	Male	43400	36100 (35600 to 36500)	7260 (6820 to 7710)
Canada	Total	15400	14200 (14000 to 14500)	1120 (870 to 1380)
Canada	Female	12800	12200 (12100 to 12300)	625 (496 to 754)
Canada	Male	17900	16300 (16100 to 16500)	1640 (1420 to 1850)
Switzerland	Total	13500	12400 (12000 to 12900)	1110 (682 to 1520)
Switzerland	Female	11800	11100 (10900 to 11300)	723 (537 to 914)
Switzerland	Male	15300	13800 (13400 to 14100)	1520 (1160 to 1870)
Chile	Total	14500	12800 (12200 to 13300)	1740 (1160 to 2310)
Chile	Female	11500	10400 (10100 to 10800)	1060 (701 to 1430)
Chile	Male	17600	15200 (14700 to 15600)	2430 (1990 to 2870)
Czech Republic	Total	21900	19500 (19100 to 19800)	2380 (2020 to 2730)
Czech Republic	Female	17800	16000 (15700 to 16200)	1810 (1540 to 2080)
Czech Republic	Male	26100	23100 (22900 to 23300)	2970 (2740 to 3200)
Germany	Total	19300	18700 (18000 to 19400)	570 (-146 to 1290)
Germany	Female	16400	16200 (15600 to 16700)	256 (-303 to 847)
Germany	Male	22200	21300 (20800 to 21700)	893 (438 to 1340)
Denmark	Total	16000	15800 (15500 to 16100)	138 (-126 to 407)
Denmark	Female	13800	13700 (13500 to 13900)	160 (-29 to 350)
Denmark	Male	18100	18000 (17800 to 18200)	115 (-76 to 303)
England & Wales	Total	17100	15400 (15000 to 15800)	1670 (1300 to 2060)
England & Wales	Female	14800	13600 (13400 to 13900)	1210 (944 to 1470)
England & Wales	Male	19300	17200 (16900 to 17500)	2140 (1880 to 2410)
Spain	Total	15800	13900 (13500 to 14300)	1920 (1500 to 2340)
Spain	Female	13000	11400 (11100 to 11700)	1560 (1260 to 1870)
Spain	Male	18800	16500 (16200 to 16800)	2280 (1990 to 2580)
Estonia	Total	22400	21100 (20400 to 21700)	1330 (676 to 1980)
Estonia	Female	17900	16900 (16800 to 17100)	933 (766 to 1100)
Estonia	Male	27400	25600 (25100 to 26200)	1790 (1220 to 2370)
Finland	Total	16700	16400 (16100 to 16600)	315 (84 to 545)
Finland	Female	13600	13500 (13400 to 13700)	74 (-77 to 228)
Finland	Male	19800	19300 (19100 to 19400)	561 (385 to 737)
France	Total	15900	15000 (14700 to 15200)	971 (743 to 1210)
France	Female	12800	12100 (12000 to 12300)	700 (540 to 856)

France	Male	19200	18000 (17800 to 18200)	1260 (1090 to 1430)
Greece	Total	18900	18100 (17700 to 18600)	796 (340 to 1260)
Greece	Female	15600	15000 (14600 to 15300)	649 (318 to 1000)
Greece	Male	22400	21400 (21100 to 21700)	954 (655 to 1250)
Croatia	Total	24700	22700 (22100 to 23300)	1980 (1380 to 2580)
Croatia	Female	20600	19000 (18500 to 19400)	1600 (1160 to 2070)
Croatia	Male	29100	26700 (26300 to 27200)	2400 (1960 to 2830)
Hungary	Total	28100	25800 (25400 to 26200)	2320 (1910 to 2740)
Hungary	Female	23600	21700 (21300 to 22000)	1920 (1590 to 2240)
Hungary	Male	33100	30300 (30100 to 30600)	2770 (2490 to 3040)
Iceland	Total	11400	11000 (10200 to 11800)	309 (-492 to 1110)
Iceland	Female	9970	9810 (9330 to 10300)	162 (-372 to 641)
Iceland	Male	12700	12200 (11600 to 12900)	455 (-186 to 1080)
Israel	Total	9660	9370 (9200 to 9550)	288 (110 to 462)
Israel	Female	8150	8080 (8000 to 8160)	71 (-11 to 154)
Israel	Male	11200	10700 (10500 to 10800)	510 (365 to 655)
Italy	Total	18000	15500 (14900 to 16200)	2460 (1830 to 3090)
Italy	Female	15500	13700 (13300 to 14200)	1800 (1340 to 2260)
Italy	Male	20600	17400 (17000 to 17900)	3150 (2720 to 3580)
South Korea	Total	11400	11500 (11200 to 11700)	-37 (-284 to 210)
South Korea	Female	8600	8640 (8490 to 8790)	-43 (-194 to 109)
South Korea	Male	14300	14300 (14100 to 14500)	-31 (-223 to 162)
Lithuania	Total	30400	26500 (25700 to 27200)	3940 (3200 to 4680)
Lithuania	Female	23100	20400 (20100 to 20800)	2640 (2310 to 2980)
Lithuania	Male	38800	33400 (32700 to 34000)	5430 (4750 to 6070)
Luxembourg	Total	12700	12200 (11700 to 12700)	531 (8 to 1050)
Luxembourg	Female	10600	10100 (9740 to 10400)	568 (241 to 877)
Luxembourg	Male	14800	14300 (13900 to 14700)	507 (83 to 947)
Latvia	Total	29400	28200 (27600 to 28900)	1150 (481 to 1810)
Latvia	Female	23300	22500 (22100 to 22900)	768 (376 to 1160)
Latvia	Male	36500	34900 (34300 to 35400)	1620 (1100 to 2170)
Northern Ireland	Total	16700	15300 (14700 to 15900)	1350 (733 to 1980)
Northern Ireland	Female	14700	13800 (13500 to 14100)	932 (604 to 1250)
Northern Ireland	Male	18700	16900 (16400 to 17400)	1780 (1290 to 2280)
Netherlands	Total	16100	14700 (14500 to 15000)	1330 (1090 to 1580)
Netherlands	Female	14400	13500 (13300 to 13600)	924 (768 to 1070)
Netherlands	Male	17800	16000 (15800 to 16200)	1780 (1580 to 1980)
Norway	Total	12300	12400 (12200 to 12500)	-61 (-197 to 74)
Norway	Female	11000	11000 (10900 to 11100)	-47 (-140 to 47)
Norway	Male	13600	13700 (13600 to 13800)	-75 (-169 to 16)
New Zealand	Total	12400	13100 (12800 to 13400)	-713 (-1020 to -408)
New Zealand	Female	10600	11200 (11000 to 11500)	-609 (-824 to -391)
New Zealand	Male	14200	15000 (14800 to 15300)	-807 (-1030 to -583)
Poland	Total	24300	21500 (21100 to 21900)	2800 (2430 to 3170)
Poland	Female	18300	16400 (16200 to 16600)	1830 (1630 to 2040)
Poland	Male	30800	26900 (26700 to 27200)	3830 (3540 to 4120)

Portugal	Total	19000	17400 (17100 to 17700)	1590 (1260 to 1920)
Portugal	Female	15200	14000 (13700 to 14200)	1260 (995 to 1530)
Portugal	Male	23200	21200 (21000 to 21400)	1950 (1760 to 2150)
Russia	Total	34300	28500 (28000 to 29100)	5810 (5280 to 6340)
Russia	Female	26000	21300 (21100 to 21500)	4760 (4530 to 4990)
Russia	Male	43900	36900 (36400 to 37400)	7020 (6550 to 7480)
Scotland	Total	21500	19800 (19200 to 20400)	1710 (1140 to 2260)
Scotland	Female	18500	17500 (17200 to 17800)	925 (613 to 1240)
Scotland	Male	24700	22100 (21700 to 22600)	2540 (2100 to 3000)
Slovakia	Total	22200	20400 (20000 to 20800)	1850 (1470 to 2230)
Slovakia	Female	17600	16100 (15900 to 16300)	1540 (1300 to 1780)
Slovakia	Male	27100	24900 (24600 to 25200)	2180 (1900 to 2460)
Slovenia	Total	18700	16800 (16300 to 17200)	1910 (1500 to 2340)
Slovenia	Female	15700	13900 (13600 to 14100)	1810 (1560 to 2060)
Slovenia	Male	21700	19700 (19300 to 20000)	2020 (1690 to 2360)
Sweden	Total	14500	13400 (13200 to 13600)	1140 (938 to 1340)
Sweden	Female	12800	12100 (11900 to 12200)	772 (640 to 901)
Sweden	Male	16200	14700 (14600 to 14900)	1510 (1360 to 1660)
Taiwan	Total	15200	15600 (15300 to 15900)	-410 (-722 to -92)
Taiwan	Female	10900	11200 (11000 to 11300)	-247 (-413 to -81)
Taiwan	Male	19600	20100 (19900 to 20400)	-572 (-842 to -312)
United States	Total	21800	18400 (18200 to 18600)	3380 (3160 to 3610)
United States	Female	17800	15300 (15200 to 15500)	2430 (2320 to 2550)
United States	Male	25900	21600 (21400 to 21800)	4350 (4170 to 4530)

Supplementary Table S4: Years of life lost per 100,000 in people <65 compared with people ≥65 years

Country/ region	Total		Female		Male	
	<65y	≥65y	<65y	≥65y	<65y	≥65y
Russia	3290 (2780 to 3810)	19600 (17800 to 21500)	2340 (2180 to 2520)	14900 (14000 to 15900)	4290 (3830 to 4740)	29000 (27300 to 30800)
Bulgaria	2650 (2220 to 3070)	15600 (11300 to 20000)	1650 (1430 to 1860)	9960 (6110 to 13900)	3630 (3260 to 3990)	24000 (22000 to 25800)
Lithuania	2580 (1790 to 3410)	9400 (7770 to 11100)	1440 (1150 to 1720)	6330 (5280 to 7350)	3740 (2990 to 4490)	15400 (14100 to 16900)
United States	2390 (2280 to 2510)	8260 (7040 to 9510)	1430 (1380 to 1480)	6910 (6300 to 7470)	3350 (3250 to 3450)	9940 (8900 to 11000)
Estonia	1340 (750 to 1930)	1300 (-863 to 3510)	931 (832 to 1030)	941 (349 to 1550)	1750 (1200 to 2310)	2020 (-190 to 4240)
Scotland	1310 (954 to 1670)	3380 (956 to 5840)	617 (413 to 826)	2100 (762 to 3430)	2010 (1720 to 2310)	4940 (2810 to 7000)
Chile	1160 (733 to 1620)	5760 (2310 to 9200)	646 (386 to 909)	3640 (1640 to 5710)	1680 (1320 to 2040)	8560 (5760 to 11400)
Canada	1020 (892 to 1150)	1600 (298 to 2920)	472 (403 to 540)	1270 (626 to 1900)	1560 (1450 to 1680)	2000 (814 to 3150)
Hungary	808 (445 to 1180)	8430 (6990 to 9820)	427 (178 to 678)	6750 (5600 to 7940)	1190 (913 to 1470)	11200 (10300 to 12000)
Poland	777 (477 to 1080)	12000 (10500 to 13500)	232 (113 to 353)	7830 (7000 to 8670)	1320 (1040 to 1580)	18200 (17000 to 19400)
England & Wales	749 (567 to 929)	5700 (3750 to 7630)	472 (353 to 592)	4170 (2940 to 5510)	1030 (891 to 1160)	7500 (6070 to 8910)
Northern Ireland	681 (345 to 1020)	4650 (1310 to 7890)	411 (268 to 557)	3310 (1620 to 4940)	953 (652 to 1260)	6290 (3500 to 9180)
Czech Republic	658 (427 to 893)	9280 (7800 to 10700)	405 (287 to 520)	6560 (5420 to 7710)	904 (706 to 1110)	13000 (12100 to 14000)
Italy	613 (301 to 908)	8560 (6000 to 11100)	288 (107 to 477)	6210 (4460 to 7930)	936 (692 to 1180)	11600 (9770 to 13400)
Slovakia	602 (329 to 873)	8100 (6260 to 9930)	411 (305 to 514)	6170 (4990 to 7360)	794 (554 to 1040)	11000 (9490 to 12500)
Iceland	571 (-150 to 1260)	-1220 (-4910 to 2710)	571 (127 to 1020)	-2050 (-4280 to 190)	566 (35.2 to 1100)	-241 (-3340 to 2800)
Latvia	555 (-114 to 1190)	3480 (1400 to 5600)	292 (35.7 to 541)	2160 (792 to 3510)	833 (274 to 1400)	6200 (4430 to 7920)
Spain	539 (293 to 775)	7510 (5590 to 9400)	328 (236 to 421)	5970 (4600 to 7330)	749 (530 to 963)	9520 (8130 to 10900)
Portugal	531 (373 to 689)	5300 (3930 to 6670)	271 (170 to 372)	4330 (3260 to 5410)	804 (684 to 926)	6660 (5820 to 7520)
Croatia	518 (159 to 873)	7490 (4930 to 10100)	341 (195 to 487)	5560 (3810 to 7320)	695 (378 to 1020)	10300 (8420 to 12300)
Finland	429 (251 to 610)	-84.6 (-890 to 716)	59.9 (-39.6 to 162)	120 (-408 to 651)	785 (640 to 937)	-345 (-990 to 283)
Austria	359 (142 to 581)	4930 (3700 to 6180)	183 (81.9 to 281)	3730 (2900 to 4560)	536 (338 to 737)	6510 (5550 to 7480)
Belgium	358 (173 to 534)	9110 (7880 to 10300)	106 (-4.47 to 216)	7670 (6730 to 8600)	606 (460 to 749)	10900 (10100 to 11800)
Sweden	224 (122 to 329)	4800 (3920 to 5700)	114 (37.6 to 192)	3170 (2610 to 3700)	333 (258 to 406)	6680 (5960 to 7390)
Netherlands	217 (85.5 to 348)	5940 (4770 to 7130)	-3.51 (-93.2 to 92.5)	4450 (3860 to 5080)	442 (344 to 539)	7790 (6780 to 8770)
Slovenia	209 (-98.2 to 510)	8600 (6880 to 10300)	94.7 (-25.7 to 220)	7480 (6470 to 8490)	322 (47.8 to 597)	10100 (8650 to 11600)
Switzerland	199 (-76.3 to 467)	5030 (3180 to 6860)	13.4 (-113 to 142)	3460 (2710 to 4230)	394 (161 to 631)	7010 (5280 to 8730)
Luxembourg	156	2730	201	2490	120	3060

	(-291 to 599)	(228 to 5210)	(-27.8 to 436)	(877 to 3990)	(-256 to 497)	(1040 to 5140)
South Korea	108	-839	198	-1180	22.6	-382
	(-58.3 to 275)	(-2130 to 482)	(122 to 274)	(-1990 to -359)	(-126 to 175)	(-1450 to 695)
Greece	107	3190	143	2240	74.6	4400
	(-55 to 270)	(1230 to 5190)	(84.7 to 202)	(788 to 3600)	(-78.1 to 230)	(3040 to 5750)
Germany	82.8	2300	-48.3	1200	210	3710
	(-248 to 419)	(-599 to 5310)	(-237 to 135)	(-1040 to 3500)	(-64 to 493)	(1790 to 5720)
France	81.4	4390	24.8	2990	142	6250
	(-57.4 to 230)	(3390 to 5370)	(-42.2 to 92.9)	(2330 to 3680)	(15.5 to 272)	(5490 to 6990)
Norway	43.2	-551	62.1	-514	25.2	-593
	(-42.2 to 132)	(-1210 to 106)	(-2.24 to 130)	(-927 to -95.6)	(-30 to 80.7)	(-1100 to -96.4)
Israel	-17.1	2540	-93.7	1160	59.1	4250
	(-121 to 88.3)	(1310 to 3780)	(-137 to -48.9)	(585 to 1730)	(-32.8 to 153)	(3160 to 5340)
New Zealand	-108	-3930	-28.3	-3520	-179	-4360
	(-282 to 58.7)	(-5720 to -2170)	(-153 to 97.5)	(-4610 to -2370)	(-295 to -62.9)	(-5710 to -3020)
Taiwan	-110	-2070	62.7	-1820	-279	-2360
	(-368 to 128)	(-3620 to -570)	(-44.1 to 165)	(-2660 to -931)	(-496 to -54.2)	(-3630 to -1100)

Supplementary Figure S3: Life expectancy at birth, 2005-2020

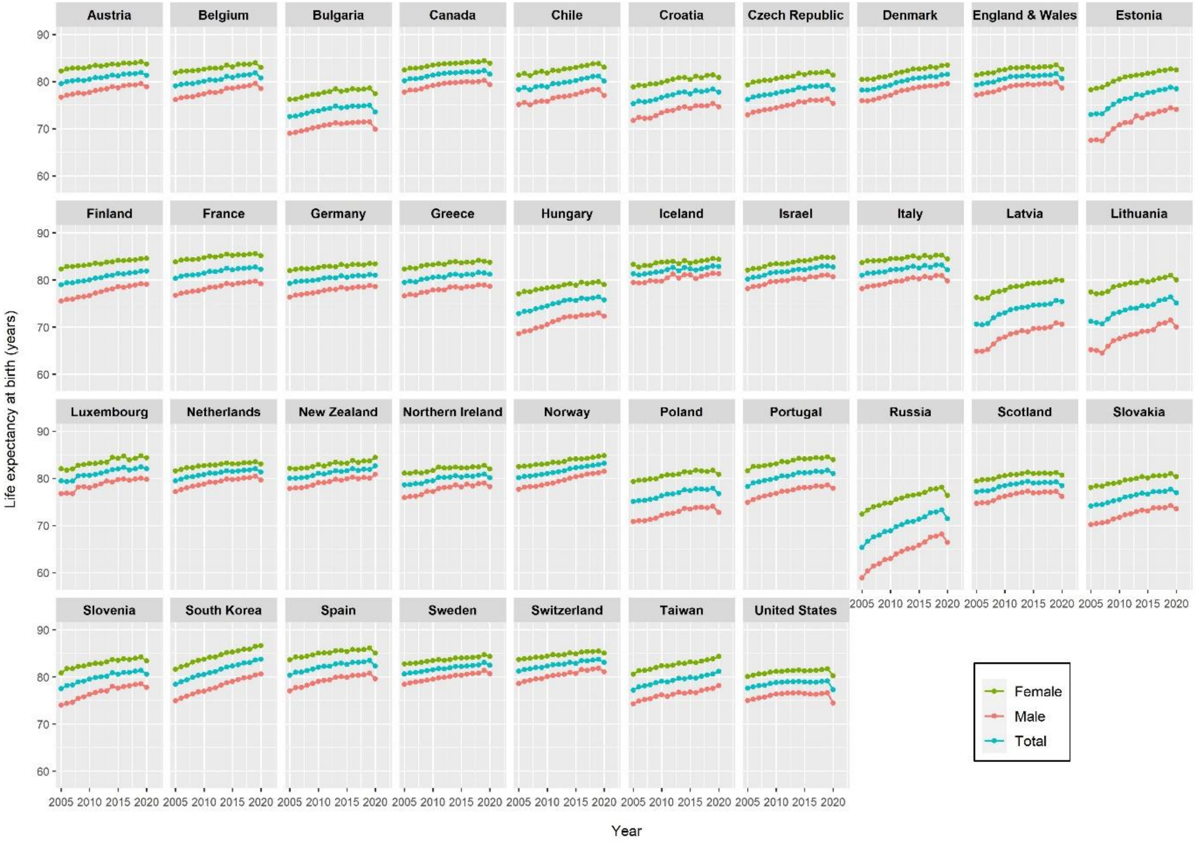

Supplementary Figure S4: Years of life lost (per 100,000) by age and sex, 2005-2020

Fig. S4(A): Men

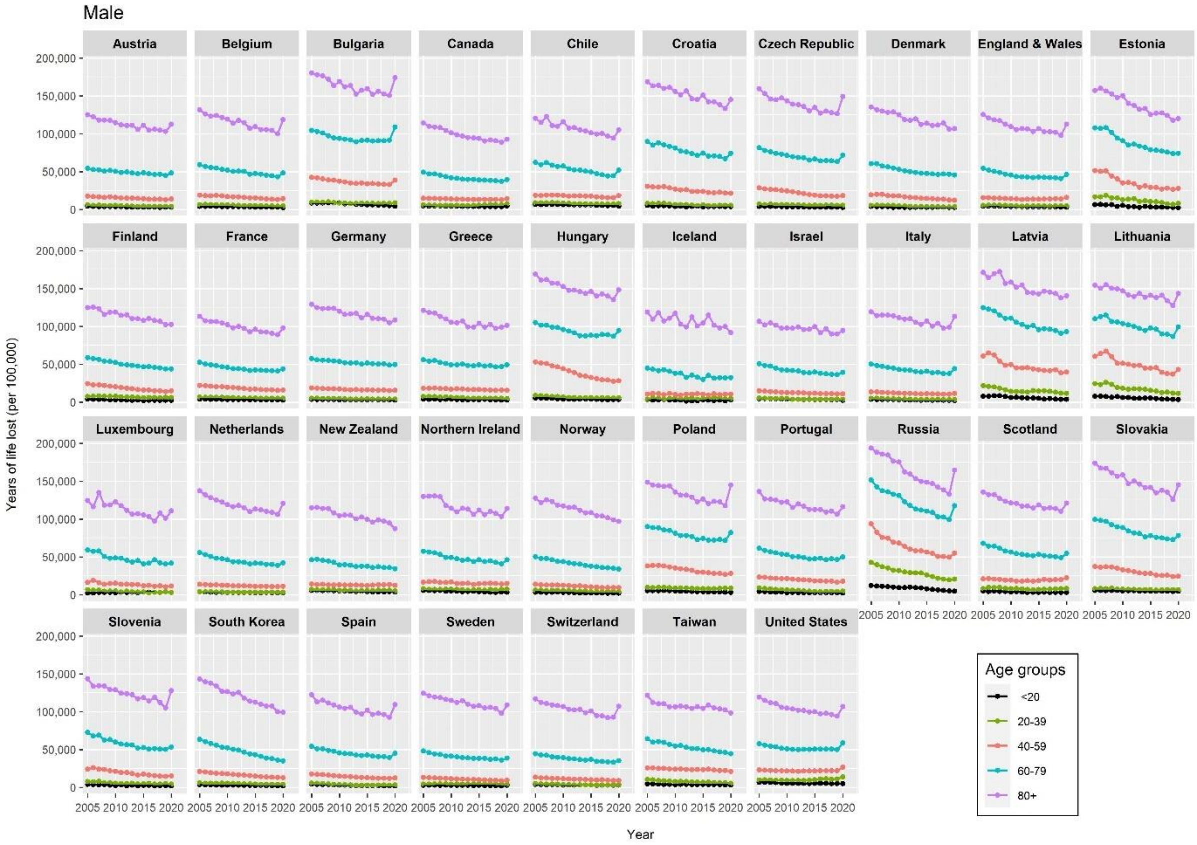

Fig. S4(B): Women

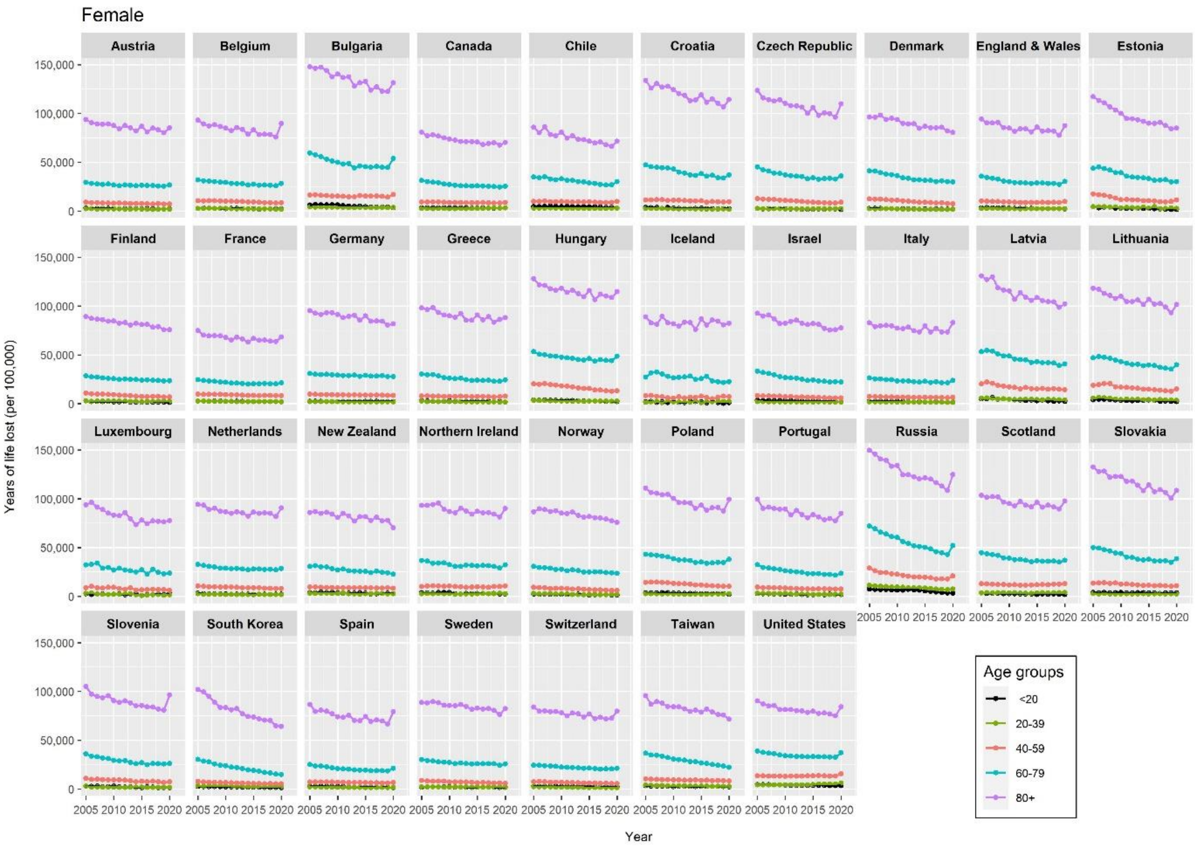

Supplementary references:

1 O'Driscoll M, Ribeiro Dos Santos G, Wang L, *et al.* Age-specific mortality and immunity patterns of SARS-CoV-2. *Nature* 2021;**590**:140–5. doi:10.1038/s41586-020-2918-0

2 Modig K, Ahlbom A, Ebeling M. Excess mortality from COVID-19: weekly excess death rates by age and sex for Sweden and its most affected region. *Eur J Public Health* 2021;**31**:17–22. doi:10.1093/eurpub/ckaa218

3 Sinnathamby MA, Whitaker H, Coughlan L, *et al.* All-cause excess mortality observed by age group and regions in the first wave of the COVID-19 pandemic in England. *Eurosurveillance* 2020;**25**:2001239. doi:10.2807/1560-7917.ES.2020.25.28.2001239

4 Preston SH, Heuveline P, Guillot M. *Demography: measuring and modeling population processes*. Malden, MA: : Blackwell Publishers 2001.

5 Coale AJ, Demeny PG, Vaughan B. *Regional model life tables and stable populations*. 2nd ed. New York: : Academic Press 1983.

6 Chiang CL. *The Life Table and Its Applications*. R.E. Krieger Publishing Company 1984.
